# Confidence intervals construction for difference of two means with incomplete correlated data

**DOI:** 10.1186/s12874-016-0125-3

**Published:** 2016-03-11

**Authors:** Hui-Qiong Li, Nian-Sheng Tang, Jie-Yi Yi

**Affiliations:** Department of Statistics, Yunnan University, No.2 Cuihu North Road, Kunming, 650091 China; School of Statistics, Beijing Normal University, No. 19 XinJieKouWai St., HaiDian District, Beijing, China

**Keywords:** Bootstrap, Confidence interval, Correlated data, Incomplete data

## Abstract

**Background:**

Incomplete data often arise in various clinical trials such as crossover trials, equivalence trials, and pre and post-test comparative studies. Various methods have been developed to construct confidence interval (CI) of risk difference or risk ratio for incomplete paired binary data. But, there is little works done on incomplete continuous correlated data. To this end, this manuscript aims to develop several approaches to construct CI of the difference of two means for incomplete continuous correlated data.

**Methods:**

Large sample method, hybrid method, simple Bootstrap-resampling method based on the maximum likelihood estimates (*B*_1_) and Ekbohm’s unbiased estimator (*B*_2_), and percentile Bootstrap-resampling method based on the maximum likelihood estimates (*B*_3_) and Ekbohm’s unbiased estimator (*B*_4_) are presented to construct CI of the difference of two means for incomplete continuous correlated data. Simulation studies are conducted to evaluate the performance of the proposed CIs in terms of empirical coverage probability, expected interval width, and mesial and distal non-coverage probabilities.

**Results:**

Empirical results show that the Bootstrap-resampling-based CIs *B*_1_, *B*_2_, *B*_4_ behave satisfactorily for small to moderate sample sizes in the sense that their coverage probabilities could be well controlled around the pre-specified nominal confidence level and the ratio of their mesial non-coverage probabilities to the non-coverage probabilities could be well controlled in the interval [0.4, 0.6].

**Conclusions:**

If one would like a CI with the shortest interval width, the Bootstrap-resampling-based CIs *B*_1_ is the optimal choice.

## Background

Incomplete data often arise in various research fields such as crossover trials, equivalence trials, and pre and post-test comparative studies. For instance, ([1] pp. 212) designed a crossover clinical trial to measure the onset of action of two doses of formoterol solution aerosol: 12 ug and 24 ug. In this study, twenty-four patients were randomly allocated in equal numbers to one of the six possible sequences of two treatments at a time. Each patient was received two aerosols at each of visits 2 and 4. After four weeks, researchers measured the forced expiratory volume of a second (FEV_1_) indicators for twenty-four patients. Due to the fact that researches did not consider all possible combinations of three treatments (e.g., placebo, 12 ug and 24 ug aerosols), which indicates that the missing data mechanism is missing completely at random (MCAR) thus FEV_1_ was only observed for 7 patients under both treatments (e.g., 12 ug and 24 ug aerosols), 9 patients only for 12 ug aerosol, and 8 patients only for 24 ug aerosol. The resultant data are shown in Table 1, which consist of two parts: the complete observations and the incomplete observations.

**Table 1:** FEV_1_ indicators of patients for 12 ug and 24 ug formoterol solution aerosol

12 *ug*(*x*_1_)	24 *ug*(*x*_2_)
2.250	2.700
0.925	0.900
1.010	1.270
2.100	2.150
2.500	2.450
1.750	1.725
1.370	1.120
3.400	
2.250	
1.460	
1.480	
2.050	
3.500	
2.650	
2.190	
0.840	
	1.750
	2.525
	1.080
	3.120
	3.100
	2.700
	1.870
	0.940

For the above crossover clinical trial, our main interest is to test the equivalence between 12 ug and 24 ug formoterol solution aerosols with respect to the FEV_1_ value. To this end, we can construct a (1−*α*)100 % confidence interval for the difference of two FEV_1_ values. If the resultant confidence interval (CI) lies entirely in the interval (−*δ*_0_,*δ*_0_) with *δ*_0_(>0) being some pre-specified clinical acceptable threshold, we thus could conclude the equivalence between two doses of formoterol solution aerosol at the *α* significance level. As a result, reliable CIs for the difference in the presence of incomplete data are necessary.

The problem of testing the equality and constructing CI for the difference of two correlated proportions in the presence of incomplete paired binary data has received considerable attention in past years. For example, ones can refer to [2–6] for the large sample method, and [7] for the corrected profile likelihood method. When sample size is small, [8] proposed the exact unconditional test procedure for testing equality of two correlated proportions with incomplete correlated data. Tang, Ling and Tian [9] developed the exact unconditional and approximate unconditional CIs for proportion difference in the presence of incomplete paired binary data. Lin et al. [10] presented a Bayesian method to test equality of two correlated proportions with incomplete correlated data. Li et al. [11] discussed the confidence interval construction for rate ratio in matched-pair studies with incomplete data. However, all the aforementioned methods were developed for incomplete paired binary data.

Statistical inference on the difference of two means with incomplete correlated data has received a limited attention. For example, [12] discussed the problem of testing the equality of two means with missing data on one response and recommended [13] statistic when the variances were not too different. Lin and Stivers [14] also gave a similar comparison. Lin and Stivers [15] and [12] suggested some test statistics for testing the equality of two means with incomplete data on both response. However, to our knowledge, little work has been done on CI construction for the difference of two means with incomplete correlated data under the MCAR assumption.

Inspired by [16–19], we develop several CIs for the difference of two means with incomplete correlated data under the MCAR assumption based on the large sample method, hybrid method and Bootstrap-resampling method. The presented Bootstrap-resampling CIs have not been considered in the literature related to missing observations.

The rest of this article is organized as follows. Several methods are presented to construct CIs for the difference of the two means with incomplete correlated data in Section “Methods”. Simulation studies and an example are conducted to evaluate the finite performance of the proposed CIs in terms of coverage probability, expected interval width, and mesial and distal non-coverage probabilities in Section “Results”. A brief discussion is given in Section “Discussion”. Some concluding remarks are given in Section “Conclusion”.

## Methods

Suppose that ***x***=(*x*_1_,*x*_2_)^′^ is a 2×1 vector of random variables, and follows a distribution with mean ***μ*** and covariance matrix ***Σ*** given by

$$\boldsymbol{\mu}=\left(\begin{array}{l} \mu_{1} \\ \mu_{2} \end{array} \right) \,\text{and}\,\, \vspace*{-4pt} \boldsymbol{\Sigma}=\left(\begin{array}{ll} {\sigma_{1}^{2}}&\, \rho\sigma_{1}\sigma_{2}\\ \rho\sigma_{1}\sigma_{2}& \,{\sigma_{2}^{2}} \end{array} \right),  $$

respectively. Let {(*x*_1*m*_,*x*_2*m*_):*m*=1,⋯,*n*} be *n* paired observations on *x*_1_ and *x*_2_, $\left \{x_{1,n+1}, \cdots, x_{1,n+n_{1}}\right \}$ be *n*_1_ additional observations on *x*_1_, $\left \{x_{2,n+1}, \cdots, x_{2,n+n_{2}}\right \}$ be *n*_2_ additional observations on *x*_2_. Thus, there are *n*_1_ missing observations on *x*_2_, and *n*_2_ missing observations on *x*_1_. Without loss of generality, the data may be presented as follows:

$$ \begin{array}{lll} x_{11}, \cdots, x_{1n}, & x_{1,n+1}, \cdots, x_{1,n+n_{1}},& \\ x_{21}, \cdots, x_{2n}, & &x_{2,n+1}, \cdots, x_{2,n+n_{2}}, \end{array}  $$

where (*x*_1*m*_,*x*_2*m*_) is referred to as a paired observation, while *x*_1,*n*+*j*_ and *x*_2,*n*+*k*_ are referred to as incomplete or unpaired observations. Similar to [20, 21], throughout this article, it is assumed that the missing data mechanism is MCAR (i.e., independent of treatment and outcome). Based on these observations, we here want to construct reliable explicit CIs for the difference of two means *δ*=*μ*_1_−*μ*_2_ under MCAR assumption.

### Confidence interval based on the large sample method

To make a comparison with the following proposed methods, we assume that ***x*** follows a bivariate normal distribution in this subsection. In this case, if only variable *x*_1_ or *x*_2_ is subject to missingness (i.e., *n*_1_=0 or *n*_2_=0), one can obtain the closed forms of the maximum likelihood estimates (MLEs) of ***μ*** and ***Σ*** [22]. However, there are no closed forms of the MLEs for ***μ*** and ***Σ*** when variables *x*_1_ and *x*_2_ are simultaneously subject to missingness (i.e., *n*_1_≠0 and *n*_2_≠0), though one can find the MLEs of ***μ*** and ***Σ*** using an iterative algorithm [23]. To get the closed forms of MLEs for ***μ*** and ***Σ***, [15] proposed the modified MLEs using a non-iterative procedure and provided several test statistics based on the obtained estimators of ***μ*** and ***Σ***.

*(i) Confidence interval based on Lin and Stivers’s test statistics*

Let $\hat {\delta }=\hat {\mu }_{1}-\hat {\mu }_{2}$ be the MLE of *δ* under the bivariate normal assumption of ***x***. When ***Σ*** is known, it follows from [15] that the MLE of *δ* is

$$ \hat{\delta}=a\overline{x}_{1}^{(n)}+\left(1-a\right)\overline{x}_{1}^{(n_{1})}-b\overline{x}_{2}^{(n)}-(1-b)\overline{x}_{2}^{(n_{2})},  $$

and the asymptotic variance of $\hat {\delta }$ can be expressed as

$$ \begin{aligned}\text{Var}(\hat{\delta})=~ &h\left\{\left[n+n_{2}\left(1-\rho\right)^{2}\right]\right\}{\sigma_{1}^{2}}-2n\rho\sigma_{1}\sigma_{2}\\ &\left.+\left[n+n_{1}\left(1-\rho^{2}\right)\right]{\sigma_{2}^{2}}\right\},\end{aligned}  $$

respectively, where $\overline {x}_{1}^{(n)}=\frac {1}{n}\sum _{j=1}^{n}x_{1j}$, $\overline {x}_{2}^{(n)}=\frac {1}{n}\sum _{j=1}^{n}x_{2j}$, $\overline {x}_{1}^{(n_{1})}=\frac {1}{n_{1}}\sum _{j=1}^{n_{1}}x_{1,n+j}$, $\overline {x}_{2}^{(n_{2})}=\frac {1}{n_{2}}\sum _{k=1}^{n_{2}}x_{2,n+k}$, *a*=*nh*(*n*+*n*_2_+*n*_1_*β*_21_), *b*=*nh*(*n*+*n*_1_+*n*_2_*β*_12_), *β*_21_=*ρσ*_2_/*σ*_1_, *β*_12_=*ρσ*_1_/*σ*_2_, *h*=1/{(*n*+*n*_1_)(*n*+*n*_2_)−*n*_1_*n*_2_*ρ*^2^}. An approximate 100(1−*α*) % CI of *δ* is given by $\left (\hat {\delta }-\textit {z}_{\alpha /2}\sqrt {\text {Var}(\hat {\delta })}, \hat {\delta }+\textit {z}_{\alpha /2}\sqrt {\text {Var}(\hat {\delta })}\right)$, which is denoted as *T*_*w*1_-CI.

Following [15], when ***Σ*** is unknown, the statistic for testing *H*_0_:*δ*=*δ*_0_ versus *H*_1_:*δ*≠*δ*_0_ is given by

$$ {}T_{1}\,=\,\frac{A\!\left(\overline{x}_{1}^{(n)}\!-\overline{x}_{1}^{(n_{1})}\right)\,-\,B\left(\overline{x}_{2}^{(n)}\!-\overline{x}_{2}^{(n_{2})}\right)\!+\overline{x}_{1}^{(n_{1})}\!-\overline{x}_{2}^{(n_{2})}\!-\delta_{0}}{\sqrt{V_{1}}},  $$

which is asymptotically distributed as *t*-distribution with *n* degrees of freedom under *H*_0_, where *V*_1_=[{*A*^2^/*n*+(1−*A*)^2^/*n*_1_ }*m*_1_+{ *B*^2^/*n*+(1−*B*)^2^/*n*_2_ } *m*_2_−2*ABm*_12_/*n*]/(*n*−1), *A*={*n*(*n*+*n*_2_+*n*_1_*m*_12_/*m*_1_}/{ (*n*+*n*_1_)(*n*+*n*_2_)−*n*_1_*n*_2_*r*^2^}^−1^, *B*={*n*(*n*+*n*_1_+*n*_2_*m*_12_/ *m*_2_} /{ (*n* + *n*_1_)(*n* + *n*_2_)−*n*_1_*n*_2_*r*^2^}^−1^, $m_{1}=\sum _{j=1}^{n} \left (x_{1j}-\overline {x}_{1}^{(n)}\right)^{2}$, $m_{2}=\sum _{j=1}^{n}\left (x_{2j}-\overline {x}_{2}^{(n)}\right)^{2}$, $m_{12}=\sum _{j=1}^{n}\left (x_{1j}\,-\,\overline {x}_{1}^{(n)}\right)\left (x_{2j}-\overline {x}_{2}^{(n)}\right)$, $r=m_{12}/\sqrt {m_{1}m_{2}}$. Therefore, the approximate 100(1−*α*) % CI on the basis of *T*_1_ is given by (*L*, *U*), where $L=A\left (\overline {x}_{1}^{(n)}-\overline {x}_{1}^{(n_{1})}\right)-B\left (\overline {x}_{2}^{(n)}-\overline {x}_{2}^{(n_{2})}\right)+\overline {x}_{1}^{(n_{1})}-\overline {x}_{2}^{(n_{2})}-t_{\alpha /2}(n)\sqrt {V_{1}}$, and $U=A\left (\overline {x}_{1}^{(n)}-\overline {x}_{1}^{(n_{1})}\right)-B\left (\overline {x}_{2}^{(n)}-\overline {x}_{2}^{(n_{2})}\right)+\overline {x}_{1}^{(n_{1})}-\overline {x}_{2}^{(n_{2})}+t_{\alpha /2}(n)\sqrt {V_{1}}$, which is denoted as *T*_1_-CI.

Another test statistic defined by [15] for testing *H*_0_:*δ*=*δ*_0_ versus *H*_1_:*δ*≠*δ*_0_, which is a generalization of [24] test statistic for two independent samples, is given by

$$ T_{2}=\frac{\bar{x}_{1}^{\left(n+n_{1}\right)}-\bar{x}_{2}^{\left(n+n_{2}\right)}-\delta_{0}}{\sqrt{h_{1}+h_{2}+h_{3}}},  $$

which is asymptotically distributed as *t* distribution with degrees *ν* of freedom, where $\bar {x}_{1}^{(n+n_{1})}=(n+n_{1})^{-1}\sum _{j=1}^{n+n_{1}}x_{1j}$, $\bar {x}_{2}^{(n+n_{2})}=(n+n_{2})^{-1}\sum _{j=1}^{n+n_{2}}x_{2j}$, *h*_1_=*n*{(*n*+*n*_2_)*m*_1_/(*n*+*n*_1_)+(*n*+*n*_1_)*m*_2_/(*n*+*n*_2_)−2*m*_12_}/{(*n*−1)(*n*+*n*_1_)(*n*+*n*_2_)}, *h*_2_=*n*_1_*b*_1_/{(*n*_1_−1)(*n*+*n*_1_)^2^}, *h*_3_=*n*_2_*b*_2_/{(*n*_2_−1)(*n*+*n*_2_)^2^}, $b_{1}=\sum _{j=n+1}^{n+n_{1}}\left (x_{1j}-\overline {x}_{1}^{(n_{1})}\right)^{2}$, $b_{2}=\sum _{j=n+1}^{n+n_{2}}\left (x_{2j}-\overline {x}_{2}^{(n_{1})}\right)^{2}$, and $\nu =\left (h_{1}+h_{2}+h_{3}\right)^{2}/\{{h_{1}^{2}}/(n-1)+{h_{2}^{2}}/(n_{1}-1)+{h_{3}^{2}}/(n_{2}-1)\}$. Therefore, the approximate 100(1−*α*) % CI of *δ* for statistic *T*_2_ is denoted as *T*_2_-CI.

When *σ*_1_=*σ*_2_, it follows from [15] that the statistic for testing *H*_0_:*δ*=*δ*_0_ versus *H*_1_:*δ*≠*δ*_0_ can be expressed as

$$ \begin{aligned} T_{3}=\left\{\bar{x}_{1}^{(n+n_{1})}-\bar{x}_{2}^{(n+n_{2})}-\delta_{0}\right\} \sqrt{\frac{(n+n_{1}+n_{2}-2)(n+n_{1})(n+n_{2})}{(b_{1}+c_{2})(2n-2nr+n_{1}+n_{2})}}, \end{aligned}  $$

which is asymptotically distribution as *t*-distribution with degrees *n*+*n*_1_+*n*_2_−4 of freedom. Note that when *n*_2_>*n*_1_, *b*_1_+*c*_2_ should be replaced by *b*_2_+*c*_1_. Thus, the approximate 100(1−*α*) % CI of *δ* for *T*_3_ is denoted as *T*_3_-CI, where $c_{1}=\sum _{j=1}^{n+n_{1}}\left (x_{1j}-{n+n_{1}}\sum _{j=1}^{n+n_{1}}x_{1j}\right)^{2}$, and $c_{2}=\sum _{j=1}^{n+n_{2}}\left (x_{2j}-\frac {1}{n+n_{2}}\sum _{j=1}^{n+n_{2}}x_{2j}\right)^{2}$.

Also, [12] presented the similar but simpler test statistics for testing the mean difference *δ*=*μ*_1_−*μ*_2_, which are adopted to construct CIs of *δ* as follows.

*(ii) Confidence interval based on Ekbohm’s test statistics*

Following [12], an unbiased estimator of *δ* is given by $\hat {\delta }=\bar {x}_{1}^{(n+n_{1})}-\bar {x}_{2}^{(n+n_{2})}$, and its variance is given by $\text {Var}(\hat {\delta })= \text {Var}(\hat {\mu })=\left \{(n+n_{2}){\sigma _{1}^{2}}+(n+n_{2}){\sigma _{2}^{2}}-2n\rho \sigma _{1}\sigma _{2}\right \}/\left \{(n+n_{1})(n+n_{2})\right \}$. An approximate 100(1 − *α*) % CI of *δ* can be obtained by $\left (\hat {\delta }-\textit {z}_{\alpha /2}\sqrt {\text {Var}(\hat {\delta })}, \hat {\delta }+\right.$$\left.\textit {z}_{\alpha /2}\sqrt {\text {Var}(\hat {\delta })}\right),$ which is denoted as *T*_*w*2_-CI.

When *σ*_1_=*σ*_2_, Ekbothm (1976) proposed the following statistic for testing *H*_0_: $T_{4}=(\tilde {\delta }-\delta _{0})\sqrt {\!(n\,+\,\!n_{1})(n\,+\,\!n_{2})\,-\,n_{1}n_{2}\lambda ^{2}}/$$ \left \{\hat \sigma \!\sqrt {2n(1-\lambda)+(n_{1}+n_{2})(1-\lambda ^{2})}\right \}$, where $\tilde {\delta }=\left [n\left (n+n_{2}+n_{1}\lambda \right)\overline {x}_{1}^{(n)}\!-n\left (n+n_{1}+n_{2}\lambda \right)\overline {x}_{2}^{(n)}+n_{1}\!\left \{n\,+\,n_{2}\!\left (1\,-\,\lambda ^{2}\right)-\!n\lambda \right \}\overline {x}_{1}^{(n_{1})}-n_{2}\left \{n\,+\,n_{1}\!\left (1\!\,-\,\!\lambda \right)^{2}\!\,-\,n\lambda \right \}\overline {x}_{2}^{(n_{2})}\right ]\!\big /\!\!\left \{(n\,+\,n_{1})(n+n_{2})\,-\,n_{1}n_{2}\lambda ^{2}\right \}$, $\hat {\sigma }^{2}\,=\,\left \{m_{1}\,+\,m_{2}\,+\,(1+\lambda ^{2})(b_{1}\,+\,b_{2})\right \}/\left \{2(n-\!1)+\!(1\,+\,\lambda ^{2})(n_{1}\,+\,n_{2}\,-\,2)\right \}$, and *λ*=2*m*_12_/(*m*_1_+*m*_2_). Under *H*_0_, *T*_4_ is asymptotically distributed as *t*-distribution with degrees *n* of freedom. Therefore, the approximate 100(1−*α*) % CI is denoted as *T*_4_-CI.

Following [12], when *σ*_1_=*σ*_2_, another statistic for testing *H*_0_ can be expressed as $T_{5}= \left (\bar {x}_{1}^{(n+n_{1})}\!-\bar {x}_{2}^{(n+n_{2})}\,-\,\delta _{0}\!\right)\!\sqrt {(n\,+\,n_{1})(n\,+\,n_{2})/(R_{1}\!\,+\,R_{2})}$, which is asymptotically distributed as *t* distribution with degrees *ν*_*σ*_ of freedom under *H*_0_, where *R*_1_ = *n*(*m*_1_ + *m*_2_ − 2*m*_12_) /(*n* − 1), *R*_2_ =(*n*_1_ + *n*_2_)(*b*_1_+*b*_2_)/(*n*_1_+*n*_2_−2), and $\nu _{\sigma }=\left (R_{1}+R_{2}\right)^{2}\!\!\!~/\left \{{R_{1}^{2}}/(n+1)+{R_{2}^{2}}/(n_{1}+n_{2})\right \}-2$. Thus, an approximate 100(1−*α*) % CI of *δ* for *T*_5_ is denoted as *T*_5_-CI.

### Confidence interval based on the generalized estimating equations(GEEs)

To relax the bivariate normality assumption of ***x***, the method of the generalized estimating equations (GEEs) with exchangeable working correlation structure (e.g., [25]) can be adopted to make statistical inference on *δ* in the incomplete correlated data because the GEE approach have become one of the most widely used methods in dealing with correlated response data [26, 27]. Following [28], the GEEs with exchangeable working correlation structure can be used to estimate parameter vector ***μ***; the so-called sandwich variance estimator can be used to consistently estimate the covariance matrix of ***μ***; and the ML method under a bivariate normal assumption via available paired observations is used to estimate the correlation parameter. Thus, an approximate 100(1−*α*) % CI of *δ* based on GEE method is denoted as *T*_*g*_-CI.

### Confidence interval based on the hybrid method

When the distribution function of ***x*** is unknown, a hybrid method is developed to construct CI of *δ* in this subsection. We first introduce the general concept of hybrid method. Let *θ*_1_ and *θ*_2_ be two parameters of interest. Now our main interest is to construct a 100(1−*α*) % two-sided CI (*L*,*U*) of *θ*_1_−*θ*_2_ via hybrid method. Let $\hat {\theta }_{1}$ and $\hat {\theta }_{2}$ be two estimates of *θ*_1_ and *θ*_2_, respectively; and let (*l*_1_,*u*_1_) and (*l*_2_,*u*_2_) denote two approximate 100(1−*α*) % CIs for *θ*_1_ and *θ*_2_, respectively. Under the dependent assumption on $\hat \theta _{1}$ and $\hat \theta _{2}$, it follows from the central limit theorem that the approximate two-sided 100(1−*α*) % CI of *θ*_1_−*θ*_2_ is given by (*L*,*U*), where

$${\kern100pt}L=\hat{\theta}_{1}-\hat{\theta}_{2}-z_{\alpha/2}\sqrt{\text{Var}(\hat{\theta}_{1})+\text{Var}(\hat{\theta}_{2})-2\text{Cov}(\hat\theta_{1}, \hat\theta_{2})}, $$

$${\kern100pt}U=\hat{\theta}_{1}-\hat{\theta}_{2}+z_{\alpha/2}\sqrt{\text{Var}(\hat{\theta}_{1})+\text{Var}(\hat{\theta}_{2})-2\text{Cov}(\hat\theta_{1}, \hat\theta_{2})}. $$

Because $\text {Cov}(\hat \theta _{1}, \hat \theta _{2})=\text {corr}(\hat \theta _{1}, \hat \theta _{2})\left \{\text {Var}(\hat {\theta }_{1})\text {Var}(\hat {\theta }_{2})\right \}^{1/2}$, the lower limit *L* and the upper limit *U* can be rewritten as

$$\begin{aligned} &L=\hat{\theta}_{1}-\hat{\theta}_{2}-z_{\alpha/2}\sqrt{\text{Var}(\hat{\theta}_{1})+\text{Var}(\hat{\theta}_{2})-2\text{corr}(\hat\theta_{1}, \hat\theta_{2})\left\{\text{Var}(\hat{\theta}_{1})\text{Var}(\hat{\theta}_{2})\right\}^{1/2}}\\ &U=\hat{\theta}_{1}-\hat{\theta}_{2}+z_{\alpha/2}\sqrt{\text{Var}(\hat{\theta}_{1})+\text{Var}(\hat{\theta}_{2})-2\text{corr}(\hat\theta_{1}, \hat\theta_{2})\left\{\text{Var}(\hat{\theta}_{1})\text{Var}(\hat{\theta}_{2})\right\}^{1/2}}, \end{aligned} $$

respectively. Note that (*l*_1_,*u*_1_) contains the plausible parameter values of *θ*_1_, and (*l*_2_,*u*_2_) contains the plausible parameter values for *θ*_2_. Among these plausible values for *θ*_1_ and *θ*_2_, the values closest to the minimum *L* and maximum *U* are respectively *l*_1_−*u*_2_ and *u*_1_−*l*_2_ in spirit of the score-type CI [29]. From the central limit theorem, the variance estimates can now be recovered from *θ*_1_=*l*_1_ as $\widehat {\text {Var}(\hat \theta _{1})}=(\hat \theta _{1}-l_{1})^{2}/z_{\alpha /2}^{2}$ and from *θ*_2_=*u*_2_ as $\widehat {\text {Var}(\hat \theta _{2})}=\left (u_{2}-\hat \theta _{2}\right)^{2}\!\!\!\big /z_{\alpha /2}^{2}$ for setting *L*. As a result, the lower limit *L* for *θ*_1_−*θ*_2_ is

(1) $$\begin{array}{@{}rcl@{}} \begin{aligned}L\,=\,\hat\theta_{1}\!-\hat{\theta}_{2}-\!\sqrt{\left(\hat{\theta}_{1}\!-l_{1}\right)^{2}\!\,+\,\left(u_{2}-\hat{\theta}_{2}\right)^{2}\!\,-\,2\widehat{\text{corr}}\!\left(\hat{\theta}_{1}, \hat{\theta}_{2}\right)\!\!\left(\hat{\theta}_{1}\,-\,l_{1}\right)\!\!\left(u_{2}\,-\,\hat{\theta}_{2}\right)}\end{aligned} \end{array} $$

Similarly, we can obtain

(2) $$\begin{array}{@{}rcl@{}} \begin{aligned}U\,=\,\hat{\theta}_{1}-\hat{\theta}_{2}\,+\,\sqrt{\!\left(u_{1}\!-\hat{\theta}_{1}\right)^{2}\!\,+\,\left(\hat{\theta}_{2}\,-\,l_{2}\right)^{2}\,-\,2\widehat{\text{corr}}\!\left(\hat{\theta}_{1}, \hat{\theta}_{2}\right)\!\!\left(u_{1}\!-\hat{\theta}_{1}\right)\!\!\left(\hat{\theta}_{2}\,-\,l_{2}\right)}\end{aligned} \end{array} $$

To obtain the above presented approximate 100(1−*α*) % hybrid CI for *μ*_1_−*μ*_2_, one requires evaluating the (1−*α*) 100 % CIs of *θ*_1_ = *μ*_1_ (denoted as (*l*_1_, *u*_1_)) and *θ*_2_=*μ*_2_ (denoted as (*l*_2_, *u*_2_)), and estimating the correlation coefficient $\widehat {\text {corr}}(\hat {\theta }_{1}, \hat \theta _{2})$. For the former, following [19], we consider the following two methods for getting the confidence limits (*l*_1_, *u*_1_) and (*l*_2_, *u*_2_) of *θ*_1_ and *θ*_2_.

(i) *The Wilson score method*

$$ l_{i}=\tilde{\theta}_{i}-\frac{z_{\alpha/2}}{N_{i}+z_{\alpha/2}^{2}}\sqrt{\frac{n}{n-1}{\sum}_{j=1}^{n}\left(x_{ij}-\hat{\theta}_{i}\right)^{2}+\frac{z_{\alpha/2}^{2}}{4}},  $$

$$ u_{i}=\tilde{\theta}_{i}+\frac{z_{\alpha/2}}{N_{i}+z_{\alpha/2}^{2}}\sqrt{\frac{n}{n-1}{\sum}_{j=1}^{n}\left(x_{ij}-\hat{\theta}_{i}\right)^{2}+\frac{z_{\alpha/2}^{2}}{4}},  $$

where *N*_*i*_=*n*+*n*_*i*_ and $\hat {\theta }_{i}=\frac {1}{N_{i}}\sum _{j=1}^{N_{i}}x_{ij}$ for *i*=1,2.

(ii)*The Agresti-coull method*

$$ {\small{\begin{aligned} {\kern20pt}l_{i}=\tilde{\theta}_{i}-z_{\alpha/2}\sqrt{\frac{\sum_{j=1}^{n}(x_{ij}-\hat{\theta}_{i})^{2}}{\left(N_{i}+z_{\alpha/2}^{2}\right)(n-1)}}, \end{aligned}}}  $$

$$\kern20pt {\small{\begin{aligned} u_{i}=\tilde{\theta}_{i}+z_{\alpha/2}\sqrt{\frac{\sum_{j=1}^{n}(x_{ij}-\hat{\theta}_{i})^{2}}{\left(N_{i}+z_{\alpha/2}^{2}\right)(n-1)}}, \end{aligned}}}  $$

where *N*_*i*_=*n*+*n*_*i*_ and $\tilde {\theta }_{i}=\left (\sum _{j=1}^{N_{i}}x_{ij}+0.5z_{\alpha /2}^{2}\right)/\left (N_{i}+z_{\alpha /2}^{2}\right)$ for *i*=1,2.

To construct CI for *δ*=*μ*_1_−*μ*_2_ via the above described hybrid method, we can simply set *θ*_1_=*μ*_1_ and *θ*_2_=*μ*_2_. If *Σ* is known, the estimated correlation coefficient $\widehat {\text {corr}}(\hat {\mu }_{1}, \hat {\mu }_{2})$ of $\hat {\mu }_{1}$ and $\hat {\mu }_{2}$ is given by $\widehat {\text {corr}}(\hat {\mu }_{1}, \hat {\mu }_{2})=2n\rho /\sqrt {(n+n_{1})(n+n_{2})}$. If *Σ* is unknown, $\widehat {\text {corr}}(\hat {\mu }_{1}, \hat {\mu }_{2})$ is given by $\widehat {\text {corr}}(\hat {\mu }_{1}, \hat {\mu }_{2})=nr/\left \{(n+n_{1})(n+n_{2})-n_{1}n_{2}r^{2}\right \}$, where $r=m_{12}/\sqrt {m_{1}m_{2}}$, $m_{1}=\sum _{j=1}^{n}\left (x_{1j}-\overline {x}_{1}^{(n)}\right)^{2}$ and $m_{2}=\sum _{j=1}^{n}\left (x_{2j}-\overline {x}_{2}^{(n)}\right)^{2}.$ Thus, using Eqs. (1) and (2) yields CIs of *δ*=*μ*_1_−*μ*_2_. When *l*_*i*_ and *u*_*i*_ are estimated by the Wilson score method, we denote the corresponding CI as *W*_*s*_-CI; when *l*_*i*_ and *u*_*i*_ are estimated by the Agresti-coull method, the corresponding CI is denoted as *W*_*a*_-CI.

#### Bootstrap-resampling-based confidence intervals

When the distribution of ***x*** is known, one can obtain the approximate CIs of *δ* based on the asymptotic distributions of the constructed test statistics under the null hypotheses *H*_0_:*δ*=*δ*_0_. However, when the distribution of ***x*** is unknown, the asymptotic distributions of the constructed test statistics may not be reliable, especially with small sample size. On the other hand, estimators of some nuisance parameters have not the closed-form solutions even if the approximate distribution is reliable, and they must be obtained by using some iterative algorithms, which are computationally intensive. In this case, the Bootstrap method is often adopted to construct CIs of parameter of interest. The Bootstrap CIs can be constructed via the following steps.

*Step 1.* Given the paired observations and incomplete observations

$${} D=\left(\!\! \begin{array}{lll} x_{11}, \cdots, x_{1n}, & x_{1,n+\!1}, \cdots, x_{1,n+n_{1}},& \\ x_{21}, \cdots, x_{2n}, & &\!x_{2,n+\!1}, \cdots, x_{2,n+n_{2}} \end{array} \!\!\right)$$

we draw *n* paired observations $\left \{(x_{1m}^{*},x_{2m}^{*}):\! m=1, \cdots,n\right \}$ with replacement from *n* paired observations {(*x*_11_,*x*_21_),⋯,(*x*_1*n*_,*x*_2*n*_)}, generate *n*_1_ observations $\{x_{1,n+j}^{*}: j=1, \cdots, n_{1}\}$ with replacement from $\left \{x_{1,n+1}, \cdots, x_{1,n+n_{1}}\right \}$, and sample *n*_2_ observations $\left \{x_{2,n+k}^{*}: k=1, \cdots, n_{2}\right \}$ with replacement from $\left \{x_{2,n+1}, \cdots, x_{2,n+n_{1}}\right \}$. Thus, we obtain the following Bootstrap resampling sample

$$D_{b}^{*}\,=\,\left(\! \begin{array}{lll} x_{11}^{*}, \cdots, x_{1n}^{*}, & x_{1,n+\!1}^{*}, \cdots, x_{1,n+n_{1}}^{*},& \\ x_{21}^{*}, \cdots, x_{2n}^{*}, & &,x_{2,n+\!1}^{*}, \cdots, x_{2,n+n_{2}}^{*} \end{array} \!\right). $$

*Step 2.* For the above generated Bootstrap resampling sample $D_{b}^{*}$, we first compute $\hat {\mu }_{1}^{*}=(n+n_{1})^{-1}\sum _{j=1}^{n+n_{1}}x_{1j}^{*}$ and $\hat {\mu }_{2}^{*}=(n+n_{2})^{-1}\sum _{j=1}^{n+n_{2}}x_{2j}^{*}$, and then calculate the estimated value $\hat {\delta }^{*}$ of *δ* via $\hat {\delta }^{*}=\hat {\mu }_{1}^{*}-\hat {\mu }_{2}^{*}$.

*Step 3.* Repeating the above steps 1 and 2 for a total of *G* times yields *G* Bootstrap estimates $\left \{\hat {\delta }_{g}^{*}: g=1,2,\cdots,G\right \}$ of *δ*. Let $\hat \delta _{(1)}^{*}<\hat \delta _{(2)}<\cdots <\hat \delta _{(G)}^{*}$ be the ordered values of $\left \{\hat \delta _{g}^{*}: g=1,2,\cdots,G\right \}$.

*Step 4.* Based on the bootstrap estimates $\left \{\hat {\delta }_{g}^{*}, g=1,2,\ldots,G\vphantom {\left \{\hat {\delta }_{g}^{*}, g=\right.}\right \}$, Bootstrap-resampling-based CIs for *δ* can be constructed as follows.

Generally, the standard error se $(\hat {\delta })$ of $\hat {\delta }$ can be estimated by the sample standard deviation of the *G* replications, i.e., $\hat {\text {se}}(\hat {\delta })=\sqrt {(G-1)^{-1}\sum _{g=1}^{G}\left (\hat {\delta }_{g}^{*}-\bar {\delta }_{B}^{*}\right)^{2}}$, where $\bar \delta _{B}^{*}=\left (\hat {\delta }_{1}^{*}+\cdots +\hat {\delta }_{G}^{*}\right)/G$. If $\left \{\hat {\delta }_{g}^{*}: g=1,\cdots, G\right \}$ is approximately normally distributed, an approximate 100(1−*α*) % Bootstrap CI for *δ* is given by $\left (\hat {\delta }-z_{\alpha /2}\hat {\text {se}}(\hat {\delta }), \hat {\delta }+z_{\alpha /2}\hat {\text {se}}(\hat \delta)\right)$, where *z*_*α*/2_ is the upper *α*/2-percentile of the standard normal distribution, which is referred as the simple Bootstrap confidence interval. When $\hat {\delta }=a\overline {x}_{1}^{(n)}+(1-a)\overline {x}_{1}^{(n_{1})}-b\overline {x}_{2}^{(n)}-(1-b)\overline {x}_{2}^{(n_{2})}$, the corresponding simple Bootstrap CI is denoted as *B*_1_. When $\hat {\delta }=\bar {x}_{1}^{(n+n_{1})}-\bar {x}_{2}^{(n+n_{2})}$, the corresponding simple Bootstrap CI is denoted as *B*_2_.

Alternatively, if $\left \{\hat {\delta }_{g}^{*}: g=1,\cdots, G\right \}$ is not normally distributed, it follows from ([16] p.132) that the approximate 100(1−*α*) % Bootstrap-resampling-based percentile CI for *δ* is $\left (\hat \delta _{\left ([G\alpha /2]\right)}^{*},\hat {\delta }_{([G(1-\alpha /2)])}^{*}\right)$, where [ *a*] represents the integer part of *a*, which is referred as the percentile Bootstrap CI. When $\hat \delta =a\overline {x}_{1}^{(n)}+(1-a)\overline {x}_{1}^{(n_{1})}-b\overline {x}_{2}^{(n)}-\left (1-b\right)\overline {x}_{2}^{(n_{2})}$, the corresponding percentile Bootstrap CI is denoted as *B*_3_. When $\hat {\delta }=\bar {x}_{1}^{(n+n_{1})}-\bar {x}_{2}^{(n+n_{2})}$, the corresponding percentile Bootstrap CI is denoted as *B*_4_.

## Results

### Simulation studies

In this subsection, we investigate the finite performance of various CIs in terms of empirical coverage probability (ECP), empirical confidence widths (ECW), and distal and mesial non-coverage probabilities (DNP and MNP) in various parameter settings via Monte Carlo simulation studies. A summary of abbreviation for various confidence intervals is presented in Table 2.

**Table 2:** Summary of various abbreviations

Abbreviation	Definition
*T*_1_	CI based on *T*_1_ statistic
*T*_2_	CI based on *T*_2_ statistic
*T*_3_	CI based on *T*_3_ statistic
*T*_4_	CI based on *T*_4_ statistic
*T*_5_	CI based on *T*_5_ statistic
*T*_*g*_	CI based on GEE method
*W*_*s*_	CI based on Wilson score method
*W*_*a*_	CI based on Agresti-coull method
*B*_1_	Simple Bootstrap CI based on
	$\hat \delta =a\overline {x}_{1}^{(n)}+\left (1-a\right)\overline {x}_{1}^{(n_{1})}-b\overline {x}_{2}^{(n)}-(1-b)\overline {x}_{2}^{(n_{2})}$
*B*_2_	Simple Bootstrap CI based on $\hat {\delta }=\bar {x}_{1}^{(n+n_{1})}-\bar {x}_{2}^{(n+n_{2})}$
*B*_3_	Percentile Bootstrap CI based on
	$\hat \delta =a\overline {x}_{1}^{(n)}+(1-a)\overline {x}_{1}^{(n_{1})}-b\overline {x}_{2}^{(n)}-(1-b)\overline {x}_{2}^{(n_{2})}$
*B*_4_	Percentile Bootstrap CI based on $\hat {\delta }=\bar {x}_{1}^{(n+n_{1})}-\bar {x}_{2}^{(n+n_{2})}$
ECPs	Empirical coverage probabilities, is defined by Eq. (3)
ECW	Empirical confidence widths, is defined by Eq. (3)
RNCP	The ratio of the mesial non-coverage probabilities to the
	non-coverage probabilities, is defined by Eqs. (4) and (5)

In the first simulation study, we consider the following case that (*n*,*n*_1_,*n*_2_) is set to be (5,2,2); *μ*_1_=0,1,2; *μ*_2_=0.25,1,1.5; *ρ*=−0.9,−0.5,−0.1,0,0.1,0.5,0.9; *δ*=*μ*_1_−*μ*_2_=−0.25,0,0.5; ${\sigma _{2}^{2}}=4$; ${\sigma _{1}^{2}}=1,8$ and *α*=0.05. For a given combination (*n*,*n*_1_,*n*_2_,*μ*_1_,*μ*_2_,*ρ*,*σ*_1_,*σ*_2_), we generate *n*+*n*_1_+*n*_2_ random samples of (*x*_1_,*x*_2_)^′^ from a bivariate normal distribution with ***μ***=(*μ*_1_,*μ*_2_)^′^ and

$$\Sigma=\left(\begin{array}{ll} {\sigma_{1}^{2}}& \rho\sigma_{1}\sigma_{2}\\ \rho\sigma_{1}\sigma_{2}& {\sigma_{2}^{2}} \end{array} \right). $$

Then, for the generated *n*+*n*_1_+*n*_2_ random samples, the *n*_1_ observations on *x*_2_ are deleted randomly. For the remaining paired *n*+*n*_2_ random samples, the *n*_2_ observations on *x*_1_ are deleted randomly. Thus, (*x*_1*m*_,*x*_2*m*_)^′^(*m*=1,⋯,*n*) are *n* pairs observations on (*x*_1_,*x*_2_)^′^; *x*_1,*n*+*j*_(*j*=1,⋯,*n*_1_) are *n*_1_ additional observations on *x*_1_; *x*_2,*n*+*k*_(*k*=1,⋯,*n*_2_) are *n*_2_ additional observations on *x*_2_. Based on the observation {(*x*_1*j*_,*x*_2*j*_):*m*=1,⋯,*n*}, {*x*_1,*n*+*j*_:*j*=1,⋯,*n*_1_}, {*x*_2,*n*+*k*_:*k*=1,⋯,*n*_2_}, we can draw 5000 bootstrap resampling samples. Independently repeating the above process *M*=10000 times, we can compute their corresponding ECP, ECW, MNP and DNP values. The ECP, ECW, MNP and DNP are defined by

(3) $$ \begin{aligned} \text{ECP}&=\frac{1}{M}\sum\limits_{m=1}^{M}I\left\{\delta\in \left[L\left(\boldsymbol{x}^{(m)}\right),U\left(\boldsymbol{x}^{(m)}\right)\right]\right\},\\ \text{ECW}&=\frac{1}{M}\sum\limits_{m=1}^{M}\left[U\left(\boldsymbol{x}^{(m)})\right)-L\left(\boldsymbol{x}^{(m)}\right)\right], \end{aligned}  $$

(4) $$ \begin{aligned} \text{MNP}&=\frac{1}{M}\sum\limits_{m=1}^{M}I\left\{\delta\in \left[-\infty, L\left(\boldsymbol{x}^{(m)}\right)\right]\right\},\\ \text{DNP}&=\frac{1}{M}\sum\limits_{m=1}^{M}I\left\{\delta\in \left[U\left(\boldsymbol{x}^{(m)}\right), +\infty\right]\right\}, \end{aligned}  $$

respectively, where $I\{\delta \in \mathcal {A}\}$ is an indicator function, which is 1 if $\delta \in \mathcal {A}$ and 0 otherwise. The ratio of the MNP to the non-coverage probability (NCP) is defined as

(5) $$ \text{RNCP}=\frac{\text{MNP}}{\text{NCP}}=\frac{\text{MNP}}{1.0-\text{ECP}}.  $$

Results are presented in Tables 3, 4 and 5. Also, to investigate the performance of the proposed CIs under the assumption ${\sigma _{1}^{2}}={\sigma _{2}^{2}}=\sigma ^{2}$, we calculate the corresponding results for *T*_3_, *T*_4_, *T*_5_, hybrid CIs, Bootstrap-resampling-based CIs when *σ*^2^=4 and (*n*,*n*_1_,*n*_2_)=(5,5,2), which are given in Tables 9, 10 and 11.

**Table 3:** ECPs of various confidence intervals under bivariate normal distribution with different *ρ* and *δ*, *μ*_1_, $\mu _{2} {\sigma _{1}^{2}}$ and (*n*,*n*_1_,*n*_2_)=(5,2,2) and ${\sigma _{2}^{2}}=4$

*ρ*	${\sigma _{1}^{2}}$	*δ*	*μ*_1_	*μ*_2_	*T*_1_	*T*_2_	*T*_*g*_	*W*_*s*_	*W*_*a*_	*B*_1_	*B*_2_	*B*_3_	*B*_4_
-0.9	1	-0.25	0	0.25	0.9390	0.9590	0.9370	0.9350	0.8800	0.9520	0.9560	0.9370	0.9570
		0	1	1	0.9440	0.9580	0.9470	0.9220	0.8760	0.9470	0.9490	0.9300	0.9490
		0.5	2	1.5	0.9430	0.9670	0.9530	0.9400	0.8860	0.9470	0.9480	0.9310	0.9480
	8	-0.25	0	0.25	0.9410	0.9630	0.9450	0.9180	0.8600	0.9430	0.9490	0.9340	0.9490
		0	1	1	0.9370	0.9580	0.9350	0.9240	0.8640	0.9510	0.9500	0.9390	0.9500
		0.5	2	1.5	0.9380	0.9570	0.9410	0.9240	0.8750	0.9570	0.9560	0.9470	0.9560
-0.5	1	-0.25	0	0.25	0.9440	0.9610	0.9530	0.9200	0.8570	0.9490	0.9430	0.9330	0.9420
		0	1	1	0.9420	0.9660	0.9230	0.9270	0.8660	0.9570	0.9560	0.9460	0.9550
		0.5	2	1.5	0.9460	0.9660	0.9380	0.9250	0.8640	0.9480	0.9560	0.9430	0.9540
	8	-0.25	0	0.25	0.9290	0.9590	0.9480	0.9230	0.8730	0.9470	0.9450	0.9390	0.9440
		0	1	1	0.9290	0.9560	0.9420	0.9210	0.8790	0.9460	0.9430	0.9380	0.9440
		0.5	2	1.5	0.9350	0.9690	0.9410	0.9330	0.8880	0.9520	0.9540	0.9470	0.9520
-0.1	1	-0.25	0	0.25	0.9300	0.9570	0.9500	0.9170	0.8630	0.9550	0.9500	0.9450	0.9470
		0	1	1	0.9380	0.9590	0.9450	0.9170	0.8600	0.9540	0.9500	0.9450	0.9520
		0.5	2	1.5	0.9400	0.9620	0.9440	0.9140	0.8560	0.9510	0.9460	0.9420	0.9460
	8	-0.25	0	0.25	0.9460	0.9600	0.9310	0.9050	0.8490	0.9460	0.9470	0.9440	0.9470
		0	1	1	0.9450	0.9670	0.9440	0.9150	0.8590	0.9560	0.9500	0.9480	0.9510
		0.5	2	1.5	0.9350	0.9610	0.9360	0.9150	0.8570	0.9500	0.9520	0.9440	0.9490
0	1	-0.25	0	0.25	0.9380	0.9610	0.9400	0.9330	0.8860	0.9550	0.9550	0.9530	0.9530
		0	1	1	0.9290	0.9610	0.9280	0.9200	0.8680	0.9470	0.9480	0.9470	0.9470
		0.5	2	1.5	0.9300	0.9580	0.9420	0.9230	0.8800	0.9520	0.9510	0.9500	0.9510
	8	-0.25	0	0.25	0.9210	0.9590	0.9390	0.9090	0.8400	0.9430	0.9450	0.9450	0.9450
		0	1	1	0.9240	0.9570	0.9400	0.9050	0.8520	0.9430	0.9440	0.9430	0.9430
		0.5	2	1.5	0.9360	0.9680	0.9380	0.9140	0.8540	0.9530	0.9530	0.9530	0.9520
0.1	1	-0.25	0	0.25	0.9310	0.9690	0.9480	0.9150	0.8530	0.9510	0.9510	0.9490	0.9490
		0	1	1	0.9330	0.9670	0.9440	0.9150	0.8550	0.9500	0.9500	0.9490	0.9510
		0.5	2	1.5	0.9310	0.9570	0.9490	0.9150	0.8630	0.9520	0.9520	0.9510	0.9520
	8	-0.25	0	0.25	0.9220	0.9520	0.9420	0.9190	0.8700	0.9510	0.9510	0.9520	0.9520
		0	1	1	0.9290	0.9540	0.9360	0.9210	0.8690	0.9490	0.9490	0.9470	0.9470
		0.5	2	1.5	0.9180	0.9530	0.9350	0.9340	0.8860	0.9520	0.9520	0.9500	0.9500
0.5	1	-0.25	0	0.25	0.9230	0.9530	0.9470	0.8980	0.8470	0.9540	0.9540	0.9530	0.9530
		0	1	1	0.9330	0.9620	0.9390	0.9050	0.8510	0.9440	0.9440	0.9440	0.9440
		0.5	2	1.5	0.9280	0.9640	0.9330	0.9140	0.8640	0.9520	0.9520	0.9500	0.9500
	8	-0.25	0	0.25	0.9360	0.9660	0.9420	0.9030	0.8450	0.9470	0.9470	0.9460	0.9460
		0	1	1	0.9220	0.9600	0.9350	0.9060	0.8410	0.9500	0.9500	0.9480	0.9480
		0.5	2	1.5	0.9300	0.9650	0.9500	0.9140	0.8570	0.9580	0.9580	0.9570	0.9570
0.9	1	-0.25	0	0.25	0.9190	0.9540	0.9400	0.9300	0.8710	0.9450	0.9450	0.9440	0.9430
		0	1	1	0.9390	0.9640	0.9460	0.9360	0.8870	0.9590	0.9580	0.9570	0.9580
		0.5	2	1.5	0.9240	0.9610	0.9310	0.9220	0.8760	0.9470	0.9460	0.9470	0.9470
	8	-0.25	0	0.25	0.9200	0.9590	0.9440	0.9050	0.8440	0.9440	0.9430	0.9430	0.9450
		0	1	1	0.9310	0.9620	0.9430	0.9040	0.8390	0.9450	0.9450	0.9460	0.9460
		0.5	2	1.5	0.9310	0.9620	0.9400	0.9190	0.8610	0.9530	0.9520	0.9520	0.9530

**Table 4:** ECW of various confidence intervals under bivariate normal distribution with different *ρ* and *δ*, *μ*_1_, *μ*_2_, ${\sigma _{1}^{2}}$ and (*n*,*n*_1_,*n*_2_)=(5,2,2) and ${\sigma _{2}^{2}}=4$

*ρ*	${\sigma _{1}^{2}}$	*δ*	*μ*_1_	*μ*_2_	*T*_1_	*T*_2_	*T*_*g*_	*W*_*s*_	*W*_*a*_	*B*_1_	*B*_2_	*B*_3_	*B*_4_
-0.9	1	-0.25	0	0.25	8.0510	9.8480	7.6040	4.9790	4.0830	6.5400	6.9700	6.5380	6.9700
		0	1	1	8.0980	9.8440	7.6290	4.9880	4.0930	6.5410	6.9710	6.5410	6.9710
		0.5	2	1.5	8.1690	9.7210	7.6410	5.0880	4.2070	6.5420	6.9700	6.5410	6.9680
	8	-0.25	0	0.25	10.8170	12.0750	9.6020	6.5090	5.2840	8.8020	9.1950	8.8010	9.1960
		0	1	1	10.8350	12.1090	9.5830	6.5080	5.2840	8.8030	9.1950	8.8050	9.1940
		0.5	2	1.5	10.8310	12.0670	9.5720	6.5610	5.3560	8.8080	9.2000	8.8070	9.1960
-0.5	1	-0.25	0	0.25	12.7390	14.0040	11.0300	7.6080	6.1620	10.2980	10.7370	10.2990	10.7370
		0	1	1	12.7510	14.0800	11.0500	7.6310	6.1810	10.3020	10.7410	10.3000	10.7380
		0.5	2	1.5	12.7460	14.0150	11.0120	7.6540	6.2220	10.3070	10.7470	10.3080	10.7450
	8	-0.25	0	0.25	7.9520	9.4420	7.3030	4.7520	3.8910	6.4600	6.5990	6.4620	6.6000
		0	1	1	7.9990	9.4880	7.3300	4.7760	3.9140	6.4630	6.6000	6.4630	6.6030
		0.5	2	1.5	7.9410	9.4190	7.3300	4.8830	4.0460	6.4650	6.6040	6.4630	6.6010
-0.1	1	-0.25	0	0.25	10.1230	11.1210	8.9290	6.0060	4.8870	8.2480	8.3910	8.2510	8.3940
		0	1	1	10.1150	11.2600	9.9060	6.0040	4.8850	8.2490	8.3920	8.2490	8.3930
		0.5	2	1.5	10.0550	11.1650	9.8830	6.0750	4.9860	8.2460	8.3880	8.2480	8.3890
	8	-0.25	0	0.25	11.8990	12.9260	10.2600	7.0330	5.7080	9.6020	9.7660	9.6030	9.7670
		0	1	1	11.9170	12.9540	10.2910	7.0500	5.7240	9.6030	9.7670	9.6020	9.7670
		0.5	2	1.5	11.9290	13.0050	10.2490	7.1130	5.8070	9.5990	9.7620	9.5980	9.7610
0	1	-0.25	0	0.25	7.4380	8.7970	6.9270	4.4570	3.6460	6.2020	6.2080	6.1980	6.2060
		0	1	1	7.4070	9.0290	6.9140	4.4570	3.6480	6.2100	6.2160	6.2100	6.2160
		0.5	2	1.5	7.4750	9.0040	6.9620	4.6380	3.8580	6.2020	6.2080	6.2000	6.2060
	8	-0.25	0	0.25	9.0700	10.2520	8.2140	5.4680	4.4610	7.4900	7.4970	7.4910	7.4960
		0	1	1	9.0480	10.0050	8.1310	5.4190	4.4290	7.4910	7.4980	7.4880	7.4960
		0.5	2	1.5	9.1370	10.2100	8.2110	5.5930	4.6170	7.4920	7.5000	7.4910	7.4970
0.1	1	-0.25	0	0.25	10.5430	11.8910	9.3750	6.3650	5.1880	8.6680	8.6760	8.6700	8.6770
		0	1	1	10.5330	11.7900	9.3610	6.3410	5.1710	8.6680	8.6760	8.6660	8.6740
		0.5	2	1.5	10.6010	11.7180	9.3710	6.4860	5.3310	8.6700	8.6780	8.6680	8.6770
	8	-0.25	0	0.25	7.3190	8.8790	6.8430	4.3920	3.5910	6.1080	6.1080	6.1070	6.1070
		0	1	1	7.2750	8.7620	6.8270	4.3840	3.5900	6.1090	6.1090	6.1090	6.1090
		0.5	2	1.5	7.3480	8.7970	6.8640	4.5800	3.8160	6.1070	6.1070	6.1040	6.1040
0.5	1	-0.25	0	0.25	8.7070	9.8380	7.9460	5.2650	4.3050	7.2590	7.2590	7.2570	7.2570
		0	1	1	8.7510	9.9250	7.9940	5.3100	4.3450	7.2570	7.2570	7.2540	7.2540
		0.5	2	1.5	8.8320	10.0890	8.0490	5.4970	4.5480	7.2590	7.2590	7.2590	7.2590
	8	-0.25	0	0.25	10.2360	11.4530	9.1100	6.1750	5.0390	8.3820	8.3820	8.3810	8.3810
		0	1	1	10.1380	11.2610	9.0610	6.1540	5.0260	8.3810	8.3810	8.3850	8.3850
		0.5	2	1.5	10.1020	11.3160	9.0800	6.2300	5.1320	8.3830	8.3830	8.3830	8.3830
0.9	1	-0.25	0	0.25	7.2300	8.9110	6.8140	4.3740	3.5750	6.0000	6.0070	6.0020	6.0090
		0	1	1	7.3030	8.6810	6.7940	4.3620	3.5700	5.9960	6.0020	5.9950	6.0020
		0.5	2	1.5	7.2340	8.8310	6.7930	4.5270	3.7720	5.9990	6.0060	5.9990	6.0050
	8	-0.25	0	0.25	8.4830	9.7340	7.8050	5.1900	4.2510	7.0030	7.0110	6.9980	7.0050
		0	1	1	8.4410	9.6700	7.7630	5.1400	4.2100	7.0010	7.0080	6.9970	7.0050
		0.5	2	1.5	8.4160	9.8250	7.8290	5.3240	4.4150	7.0000	7.0080	7.0020	7.0100

**Table 5:** RNCP of various confidence intervals under bivariate normal distribution with different *ρ* and *δ*, *μ*_1_, *μ*_2_, ${\sigma _{1}^{2}}$ and (*n*,*n*_1_,*n*_2_)=(5,2,2) and ${\sigma _{2}^{2}}=4$

*ρ*	${\sigma _{1}^{2}}$	*δ*	*μ*_1_	*μ*_2_	*T*_1_	*T*_2_	*T*_*g*_	*W*_*s*_	*W*_*a*_	*B*_1_	*B*_2_	*B*_3_	*B*_4_
-0.9	1	-0.25	0	0.25	0.4754	0.4805	0.4731	0.4769	0.4660	0.5000	0.4091	0.4921	0.4186
		0	1	1	0.4286	0.5286	0.4563	0.3846	0.4892	0.4528	0.4314	0.4286	0.4706
		0.5	2	1.5	0.4737	0.5909	0.4839	0.4667	0.4590	0.4906	0.4231	0.4638	0.4038
	8	-0.25	0	0.25	0.4237	0.5108	0.5048	0.4268	0.4574	0.5088	0.5686	0.5303	0.5686
		0	1	1	0.4603	0.5143	0.4857	0.4474	0.5000	0.5102	0.5000	0.5082	0.5000
		0.5	2	1.5	0.4677	0.5744	0.4545	0.5395	0.4983	0.4186	0.4545	0.4717	0.4773
-0.5	1	-0.25	0	0.25	0.5536	0.5436	0.5234	0.5375	0.5289	0.5686	0.5789	0.5821	0.5862
		0	1	1	0.5000	0.5235	0.4948	0.4795	0.5389	0.4651	0.4773	0.4815	0.4667
		0.5	2	1.5	0.5741	0.5176	0.5294	0.6533	0.5266	0.5577	0.6591	0.6140	0.6304
	8	-0.25	0	0.25	0.5070	0.5829	0.5098	0.5195	0.5481	0.5472	0.5273	0.5410	0.5357
		0	1	1	0.5352	0.5364	0.5306	0.4684	0.5585	0.5370	0.5263	0.5645	0.5536
		0.5	2	1.5	0.4769	0.5355	0.4719	0.3731	0.5256	0.5208	0.4348	0.4717	0.4375
-0.1	1	-0.25	0	0.25	0.5000	0.5744	0.5300	0.4699	0.6086	0.5333	0.5000	0.4727	0.4717
		0	1	1	0.4839	0.5585	0.4842	0.4458	0.5714	0.5000	0.5400	0.5091	0.5417
		0.5	2	1.5	0.5333	0.5632	0.5000	0.5116	0.5000	0.5102	0.5185	0.5000	0.5000
	8	-0.25	0	0.25	0.4630	0.5750	0.4848	0.4526	0.5176	0.4444	0.4151	0.4464	0.4528
		0	1	1	0.5091	0.5879	0.5104	0.5059	0.5119	0.5455	0.4800	0.5000	0.4898
		0.5	2	1.5	0.5385	0.5179	0.5288	0.5529	0.5248	0.5200	0.5208	0.5179	0.4902
0	1	-0.25	0	0.25	0.5484	0.5641	0.5667	0.6119	0.4800	0.4889	0.5333	0.5319	0.5319
		0	1	1	0.4789	0.5923	0.5000	0.4000	0.4996	0.4906	0.4808	0.4906	0.4906
		0.5	2	1.5	0.4286	0.5714	0.5000	0.2857	0.5097	0.5000	0.5102	0.5200	0.5306
	8	-0.25	0	0.25	0.4684	0.5829	0.5149	0.4835	0.5397	0.4912	0.5091	0.5091	0.5091
		0	1	1	0.5789	0.5977	0.4700	0.4737	0.5028	0.4561	0.4464	0.4912	0.4561
		0.5	2	1.5	0.5313	0.5500	0.5000	0.5233	0.5100	0.4894	0.5106	0.5106	0.5000
0.1	1	-0.25	0	0.25	0.5217	0.5065	0.5488	0.5176	0.5566	0.5102	0.5102	0.4902	0.4902
		0	1	1	0.5224	0.5788	0.4651	0.5176	0.5212	0.4200	0.4200	0.4314	0.4286
		0.5	2	1.5	0.5362	0.5116	0.5824	0.6235	0.5852	0.5417	0.5417	0.5714	0.5417
	8	-0.25	0	0.25	0.4359	0.5417	0.4490	0.5309	0.5833	0.4490	0.4490	0.4583	0.4583
		0	1	1	0.4789	0.5304	0.4904	0.3544	0.4914	0.4118	0.4118	0.4528	0.4528
		0.5	2	1.5	0.4878	0.5170	0.5053	0.2879	0.5314	0.4167	0.4167	0.4200	0.4200
0.5	1	-0.25	0	0.25	0.4935	0.5106	0.4563	0.4510	0.5125	0.5000	0.5000	0.5106	0.5106
		0	1	1	0.5522	0.5947	0.4505	0.4211	0.5085	0.3929	0.3929	0.4107	0.4107
		0.5	2	1.5	0.4861	0.5944	0.4943	0.5000	0.4692	0.5417	0.5417	0.5000	0.5000
	8	-0.25	0	0.25	0.4688	0.5647	0.4592	0.4227	0.5081	0.5472	0.5472	0.5185	0.5185
		0	1	1	0.5256	0.5750	0.5474	0.5426	0.5008	0.5200	0.5200	0.5577	0.5577
		0.5	2	1.5	0.5286	0.5000	0.4875	0.5233	0.5093	0.5238	0.5238	0.5349	0.5349
0.9	1	-0.25	0	0.25	0.5062	0.5652	0.5000	0.5714	0.4861	0.5273	0.5273	0.5357	0.5263
		0	1	1	0.5246	0.5111	0.5238	0.3281	0.5100	0.5122	0.5000	0.4884	0.5000
		0.5	2	1.5	0.4605	0.5692	0.4141	0.2179	0.2217	0.4528	0.4444	0.4340	0.4340
	8	-0.25	0	0.25	0.5250	0.5341	0.5104	0.5053	0.5045	0.5179	0.5088	0.5439	0.5455
		0	1	1	0.5362	0.5579	0.5155	0.4688	0.6133	0.5273	0.5273	0.5370	0.5370
		0.5	2	1.5	0.5217	0.5579	0.4778	0.4938	0.4672	0.4681	0.4583	0.5000	0.4681

Following [17, 30], an interval can be regarded as satisfactory if (i) its ECP is close to the pre-specified 95 % confidence level, (ii) it possesses shorter interval width, and (iii) its RNCP lies in the interval [0.4,0.6]; *too mesially located* if its RNCP is less than 0.4; and *too distally* if its RNCP is greater than 0.6.

In the second Monte Carlo simulation study, we assume that the random samples of bivariate variables *x*_1_ and *x*_2_ are generated from a bivariate *t*-distribution with five degrees of freedom, and mean ***μ*** and scale parameter *Σ* specified in the first simulation study. The corresponding results with (*n*,*n*_1_,*n*_2_)=(5,5,5) are given in Tables 6, 7 and 8. Similarly, we calculate the corresponding results for *T*_3_, *T*_4_, *T*_5_, hybrid CIs, Bootstrap-resampling-based CIs when *σ*^2^=4 and (*n*,*n*_1_,*n*_2_)=(5,5,2), which are given in Tables 9, 10 and 11.

**Table 6:** ECPs of various confidence intervals under bivariate t-distribution with different *ρ* and *δ*, *μ*_1_, *μ*_2_, ${\sigma _{1}^{2}}$ and (*n*,*n*_1_,*n*_2_)=(5,5,5) and ${\sigma _{2}^{2}}=4$

*ρ*	${\sigma _{1}^{2}}$	*δ*	*μ*_1_	*μ*_2_	*T*_1_	*T*_2_	*T*_*g*_	*W*_*s*_	*W*_*a*_	*B*_1_	*B*_2_	*B*_3_	*B*_4_
-0.9	1	-0.25	0	0.25	0.9260	0.9750	0.9460	0.9510	0.9020	0.9470	0.9470	0.9500	0.9500
		0	1	1	0.9060	0.9590	0.9490	0.9340	0.8820	0.9450	0.9450	0.9510	0.9510
		0.5	2	1.5	0.9160	0.9710	0.9370	0.9480	0.8930	0.9490	0.9490	0.9530	0.9530
	8	-0.25	0	0.25	0.8950	0.9630	0.9380	0.9460	0.8920	0.9490	0.9380	0.9410	0.9410
		0	1	1	0.9030	0.9580	0.9430	0.9450	0.9020	0.9400	0.9410	0.9410	0.9410
		0.5	2	1.5	0.9080	0.9640	0.9370	0.9490	0.9070	0.9500	0.9480	0.9520	0.9520
-0.5	1	-0.25	0	0.25	0.9160	0.9700	0.9460	0.9380	0.8810	0.9440	0.9410	0.9430	0.9420
		0	1	1	0.9150	0.9670	0.9510	0.9380	0.8970	0.9470	0.9480	0.9480	0.9480
		0.5	2	1.5	0.9190	0.9650	0.9440	0.9440	0.8940	0.9480	0.9520	0.9540	0.9540
	8	-0.25	0	0.25	0.9160	0.9680	0.9490	0.9580	0.9160	0.9530	0.9480	0.9440	0.9510
		0	1	1	0.9080	0.9690	0.9510	0.9590	0.9200	0.9460	0.9450	0.9400	0.9480
		0.5	2	1.5	0.9130	0.9750	0.9400	0.9630	0.9200	0.9410	0.9410	0.9230	0.9460
-0.1	1	-0.25	0	0.25	0.9230	0.9660	0.9480	0.9500	0.9020	0.9530	0.9470	0.9410	0.9490
		0	1	1	0.9060	0.9600	0.9380	0.9370	0.8920	0.9430	0.9450	0.9390	0.9500
		0.5	2	1.5	0.9020	0.9660	0.9410	0.9400	0.8910	0.9530	0.9460	0.9350	0.9460
	8	-0.25	0	0.25	0.9110	0.9670	0.9450	0.9650	0.9290	0.9440	0.9420	0.8800	0.9470
		0	1	1	0.9190	0.9720	0.9360	0.9650	0.9270	0.9510	0.9450	0.8810	0.9470
		0.5	2	1.5	0.9140	0.9700	0.9390	0.9630	0.9270	0.9480	0.9440	0.8890	0.9470
0	1	-0.25	0	0.25	0.9180	0.9580	0.9430	0.9500	0.8980	0.9470	0.9390	0.7900	0.9420
		0	1	1	0.9150	0.9710	0.9550	0.9550	0.9130	0.9490	0.9500	0.8030	0.9500
		0.5	2	1.5	0.9180	0.9670	0.9500	0.9590	0.9200	0.9450	0.9510	0.7940	0.9540
	8	-0.25	0	0.25	0.9380	0.9660	0.9380	0.9560	0.9280	0.9510	0.9510	0.9380	0.9530
		0	1	1	0.9360	0.9650	0.9340	0.9530	0.9220	0.9560	0.9520	0.9370	0.9540
		0.5	2	1.5	0.9310	0.9540	0.9340	0.9510	0.9230	0.9450	0.9530	0.9400	0.9540
0.1	1	-0.25	0	0.25	0.9360	0.9640	0.9420	0.9530	0.9210	0.9480	0.9510	0.9430	0.9550
		0	1	1	0.9350	0.9620	0.9340	0.9520	0.9190	0.9560	0.9520	0.9400	0.9520
		0.5	2	1.5	0.9290	0.9600	0.9340	0.9440	0.9160	0.9440	0.9470	0.9340	0.9480
	8	-0.25	0	0.25	0.9300	0.9530	0.9330	0.9470	0.9190	0.9400	0.9380	0.9350	0.9400
		0	1	1	0.9340	0.9590	0.9310	0.9520	0.9160	0.9410	0.9410	0.9360	0.9420
		0.5	2	1.5	0.9390	0.9660	0.9330	0.9520	0.9210	0.9530	0.9500	0.9490	0.9530
0.5	1	-0.25	0	0.25	0.9370	0.9640	0.9370	0.9490	0.9120	0.9450	0.9440	0.9430	0.9470
		0	1	1	0.9450	0.9590	0.9360	0.9450	0.9080	0.9460	0.9420	0.9380	0.9440
		0.5	2	1.5	0.9430	0.9680	0.9400	0.9520	0.9200	0.9540	0.9480	0.9490	0.9540
	8	-0.25	0	0.25	0.9340	0.9580	0.9460	0.9520	0.9190	0.9420	0.9450	0.9470	0.9480
		0	1	1	0.9400	0.9630	0.9470	0.9530	0.9210	0.9550	0.9560	0.9580	0.9580
		0.5	2	1.5	0.9270	0.9610	0.9330	0.9470	0.9230	0.9420	0.9420	0.9470	0.9460
0.9	1	-0.25	0	0.25	0.9430	0.9660	0.9410	0.9500	0.9140	0.9470	0.9470	0.9480	0.9480
		0	1	1	0.9410	0.9530	0.9440	0.9400	0.9040	0.9470	0.9460	0.9510	0.9500
		0.5	2	1.5	0.9430	0.9660	0.9480	0.9490	0.9160	0.9540	0.9560	0.9550	0.9560
	8	-0.25	0	0.25	0.9320	0.9540	0.9520	0.9450	0.9200	0.9460	0.9460	0.9490	0.9490
		0	1	1	0.9460	0.9660	0.9470	0.9590	0.9300	0.9470	0.9470	0.9490	0.9490
		0.5	2	1.5	0.9410	0.9580	0.9460	0.9510	0.9200	0.9550	0.9550	0.9580	0.9580

**Table 7:** ECW of various confidence intervals under bivariate t-distribution with different *ρ* and *δ*, *μ*_1_, *μ*_2_, ${\sigma _{1}^{2}}$ and (*n*,*n*_1_,*n*_2_)=(5,5,5) and ${\sigma _{2}^{2}}=4$

*ρ*	${\sigma _{1}^{2}}$	*δ*	*μ*_1_	*μ*_2_	*T*_1_	*T*_2_	*T*_*g*_	*W*_*s*_	*W*_*a*_	*B*_1_	*B*_2_	*B*_3_	*B*_4_
-0.9	1	-0.25	0	0.25	39.9860	40.8450	35.0080	27.6870	23.6020	35.9410	35.9410	36.4110	36.4110
		0	1	1	39.5890	40.3210	34.6710	27.5260	23.4670	35.9280	35.9280	36.4080	36.4080
		0.5	2	1.5	39.2290	40.6160	34.8600	27.6570	23.5830	35.9050	35.9050	36.3930	36.3930
	8	-0.25	0	0.25	32.6680	34.3430	29.0000	22.6520	19.2790	29.8250	29.8650	30.3280	30.3510
		0	1	1	32.7540	34.2080	28.8030	22.5610	19.2030	29.8370	29.8760	30.3360	30.3630
		0.5	2	1.5	32.3510	34.3530	28.9420	22.6560	19.2890	29.8380	29.8770	30.3350	30.3580
-0.5	1	-0.25	0	0.25	38.5200	39.5450	34.4200	27.2240	23.2190	35.0120	35.0610	35.4930	35.5290
		0	1	1	37.8690	39.0530	33.9970	26.9350	22.9710	35.0060	35.0530	35.4800	35.5150
		0.5	2	1.5	38.7140	39.7290	34.1600	27.1930	23.1930	35.0190	35.0680	35.4960	35.5300
	8	-0.25	0	0.25	29.3790	32.8700	27.8810	21.7710	18.5360	27.1550	28.3790	27.6280	28.8530
		0	1	1	28.9240	32.3540	27.5350	21.5080	18.3100	27.1670	28.4000	27.6550	28.8590
		0.5	2	1.5	30.1060	33.6320	28.5080	22.3350	19.0220	27.1880	28.4140	27.6570	28.8780
-0.1	1	-0.25	0	0.25	31.3890	36.1820	31.7890	25.3610	21.6710	29.6610	31.4040	30.1280	31.7960
		0	1	1	30.9880	35.1900	30.9350	24.6920	21.0960	29.6870	31.4300	30.1620	31.8160
		0.5	2	1.5	31.1860	35.2420	31.2250	24.8410	21.2380	29.6730	31.4220	30.1440	31.8080
	8	-0.25	0	0.25	23.8340	31.2500	26.6610	20.8190	17.7300	20.9570	26.8340	21.2360	27.2880
		0	1	1	23.4990	31.1470	26.6370	20.8520	17.7590	20.9660	26.8530	21.2550	27.3120
		0.5	2	1.5	23.1750	30.4390	26.1330	20.3920	17.3770	20.9520	26.8280	21.2470	27.2670
0	1	-0.25	0	0.25	16.7960	30.7840	27.3290	21.9590	18.8280	16.6250	27.2590	16.9850	27.6450
		0	1	1	17.1650	30.5510	27.3190	21.8760	18.7550	16.6250	27.2600	16.9750	27.6580
		0.5	2	1.5	16.9980	30.6500	27.2430	22.0410	18.9120	16.6160	27.2610	16.9700	27.6440
	8	-0.25	0	0.25	27.2420	29.7100	27.2280	22.7000	20.2600	26.0380	27.9560	26.2890	28.2960
		0	1	1	27.6030	29.9040	27.3850	22.8460	20.3900	26.0420	27.9600	26.2820	28.2960
		0.5	2	1.5	27.4440	29.6420	27.2230	22.6840	20.2480	26.0420	27.9660	26.2770	28.2950
0.1	1	-0.25	0	0.25	36.7630	38.5960	35.2540	29.7190	26.5230	35.0020	36.7010	35.3140	37.1130
		0	1	1	36.9580	38.9090	35.4500	29.9490	26.7290	34.9960	36.6930	35.3230	37.1390
		0.5	2	1.5	36.6820	38.7640	35.2490	29.7940	26.5910	34.9890	36.6840	35.3050	37.1090
	8	-0.25	0	0.25	26.8170	28.2480	25.9750	21.6000	19.2790	25.9390	26.5530	26.1980	26.8650
		0	1	1	27.0150	28.2910	26.0250	21.6540	19.3270	25.9380	26.5470	26.1960	26.8510
		0.5	2	1.5	27.1980	28.6990	26.3160	21.9060	19.5540	25.9400	26.5480	26.1920	26.8500
0.5	1	-0.25	0	0.25	35.0610	35.8660	32.9600	27.7210	24.7450	33.0080	33.6310	33.3300	34.0030
		0	1	1	35.2510	35.8450	32.9690	27.7590	24.7790	32.9910	33.6170	33.3000	33.9910
		0.5	2	1.5	34.6160	35.6320	32.7980	27.5930	24.6330	32.9950	33.6200	33.3110	33.9920
	8	-0.25	0	0.25	26.0830	26.9080	24.8210	20.5850	18.3740	25.0540	25.0810	25.3290	25.3590
		0	1	1	25.6840	26.7000	24.6450	20.4400	18.2450	25.0390	25.0650	25.3160	25.3480
		0.5	2	1.5	25.9800	26.9400	24.8400	20.5900	18.3810	25.0410	25.0680	25.3280	25.3580
0.9	1	-0.25	0	0.25	31.7980	32.2880	29.9420	25.1170	22.4300	30.2210	30.2530	30.5230	30.5690
		0	1	1	31.8710	32.0900	29.8060	24.9940	22.3200	30.1980	30.2290	30.5050	30.5500
		0.5	2	1.5	31.3990	32.0560	29.7450	24.9700	22.3010	30.2140	30.2440	30.5180	30.5600
	8	-0.25	0	0.25	25.4700	26.4860	24.4770	20.2660	18.0900	24.6850	24.6850	24.9600	24.9600
		0	1	1	25.5190	26.3770	24.3630	20.1840	18.0160	24.6740	24.6740	24.9450	24.9450
		0.5	2	1.5	25.4630	26.4500	24.4990	20.2850	18.1100	24.6930	24.6930	24.9760	24.9760

**Table 8:** RNCP of various confidence intervals under bivariate t-distribution with different *ρ* and *δ*, *μ*_1_, *μ*_2_, ${\sigma _{1}^{2}}$ and (*n*,*n*_1_,*n*_2_)=(5,5,5) and ${\sigma _{2}^{2}}=4$

*ρ*	${\sigma _{1}^{2}}$	*δ*	*μ*_1_	*μ*_2_	*T*_1_	*T*_2_	*T*_*g*_	*W*_*s*_	*W*_*a*_	*B*_1_	*B*_2_	*B*_3_	*B*_4_
-0.9	1	-0.25	0	0.25	0.4324	0.5200	0.5000	0.5918	0.5102	0.4717	0.4717	0.4800	0.4800
		0	1	1	0.4574	0.4634	0.5062	0.4848	0.5000	0.4727	0.4727	0.5102	0.5102
		0.5	2	1.5	0.4524	0.5862	0.5238	0.5385	0.5047	0.4118	0.4118	0.4255	0.4255
	8	-0.25	0	0.25	0.4762	0.4865	0.4878	0.4815	0.4815	0.4754	0.4677	0.4746	0.4746
		0	1	1	0.5361	0.5238	0.4675	0.5091	0.5000	0.4833	0.4746	0.4746	0.4746
		0.5	2	1.5	0.4783	0.5278	0.4795	0.5098	0.5484	0.5400	0.5000	0.5208	0.5208
-0.5	1	-0.25	0	0.25	0.4524	0.4000	0.4595	0.4839	0.4538	0.4464	0.4237	0.4211	0.4138
		0	1	1	0.6000	0.6061	0.5797	0.5645	0.5534	0.5283	0.5385	0.5577	0.5385
		0.5	2	1.5	0.5062	0.5429	0.5455	0.5357	0.5660	0.5000	0.5000	0.5435	0.5435
	8	-0.25	0	0.25	0.4762	0.5000	0.5070	0.5952	0.5119	0.5106	0.5385	0.5179	0.5306
		0	1	1	0.5217	0.5806	0.5085	0.5854	0.5500	0.4815	0.5273	0.5167	0.5000
		0.5	2	1.5	0.4943	0.4000	0.4000	0.4595	0.5125	0.5316	0.5217	0.5195	0.5313
-0.1	1	-0.25	0	0.25	0.5584	0.4706	0.5323	0.5800	0.4796	0.5319	0.5660	0.5932	0.5686
		0	1	1	0.5532	0.5500	0.5278	0.5714	0.5463	0.4737	0.4727	0.4754	0.5000
		0.5	2	1.5	0.4490	0.4706	0.4348	0.4333	0.4679	0.4255	0.4815	0.4769	0.4630
	8	-0.25	0	0.25	0.4831	0.4545	0.5231	0.4000	0.4648	0.5714	0.5000	0.4917	0.5283
		0	1	1	0.5062	0.5000	0.4844	0.4571	0.5068	0.4898	0.4727	0.4958	0.4717
		0.5	2	1.5	0.4651	0.5000	0.4590	0.5676	0.5479	0.4423	0.4464	0.4595	0.4717
0	1	-0.25	0	0.25	0.5244	0.5714	0.6140	0.5800	0.5196	0.5094	0.5246	0.4857	0.5000
		0	1	1	0.5059	0.4483	0.4444	0.4444	0.4828	0.5490	0.5600	0.5228	0.5600
		0.5	2	1.5	0.5366	0.3939	0.4800	0.4146	0.4875	0.4545	0.5306	0.5097	0.5217
	8	-0.25	0	0.25	0.5161	0.6176	0.5968	0.6136	0.5972	0.5714	0.6122	0.5968	0.5957
		0	1	1	0.4844	0.4571	0.4697	0.4468	0.4744	0.5682	0.5208	0.5079	0.5000
		0.5	2	1.5	0.4928	0.4130	0.4545	0.4286	0.4545	0.4545	0.4255	0.4833	0.4348
0.1	1	-0.25	0	0.25	0.5469	0.5833	0.5862	0.6170	0.5696	0.5385	0.5102	0.5088	0.5333
		0	1	1	0.5692	0.4737	0.5303	0.5208	0.5309	0.5227	0.5208	0.5000	0.5000
		0.5	2	1.5	0.4507	0.4500	0.4394	0.4107	0.4286	0.4464	0.4717	0.4545	0.4808
	8	-0.25	0	0.25	0.5000	0.5319	0.5373	0.5472	0.5062	0.5000	0.5161	0.5077	0.5000
		0	1	1	0.5303	0.5366	0.5072	0.5417	0.4762	0.4746	0.4915	0.4844	0.5000
		0.5	2	1.5	0.5246	0.5294	0.5373	0.5417	0.5443	0.5532	0.5400	0.5294	0.5319
0.5	1	-0.25	0	0.25	0.6190	0.5833	0.5397	0.6078	0.5341	0.5091	0.5714	0.5614	0.5660
		0	1	1	0.4545	0.4878	0.4844	0.5091	0.4565	0.4444	0.4828	0.4677	0.4643
		0.5	2	1.5	0.5088	0.5625	0.5000	0.5208	0.5125	0.5000	0.4615	0.4510	0.4565
	8	-0.25	0	0.25	0.5303	0.4762	0.5000	0.4583	0.4815	0.5172	0.5273	0.5283	0.5385
		0	1	1	0.5500	0.5676	0.5714	0.5532	0.6076	0.5333	0.5455	0.5238	0.5238
		0.5	2	1.5	0.5479	0.5385	0.5224	0.5660	0.5584	0.4655	0.4828	0.4717	0.4630
0.9	1	-0.25	0	0.25	0.5088	0.5000	0.4746	0.4800	0.4884	0.4906	0.4717	0.4808	0.4808
		0	1	1	0.4915	0.5106	0.4848	0.5000	0.4479	0.4151	0.4259	0.4286	0.4200
		0.5	2	1.5	0.5789	0.5294	0.5161	0.5098	0.5595	0.4783	0.4773	0.5333	0.5455
	8	-0.25	0	0.25	0.4559	0.5435	0.5147	0.4909	0.4875	0.5000	0.5000	0.4902	0.4902
		0	1	1	0.4815	0.5294	0.5283	0.5366	0.5429	0.6038	0.6038	0.5686	0.5686
		0.5	2	1.5	0.4407	0.4524	0.5000	0.5102	0.5250	0.4222	0.4222	0.4048	0.4048

To investigate powers for the proposed CIs, we calculated the power in both the first and second simulation study. The results are shown in Tables 12 and 13. There is very little power in both the first and second simulation study to exclude a difference of zero.

### Results of simulation studies

From Tables 3, 4, 5, 6, 7, 8, 9, 10, 11, 12 and 13, we have the following findings. First, when *Σ* is unknown, the CIs based on the the Bootstrap-resampling-based methods except for *B*_3_ behave satisfactorily in the sense that their ECPs are close to the pre-specified confidence level 95 % (e.g., see Tables 3 and 6); the CI based on the Bootstrap-resampling-based method *B*_1_ generally yielded shorter ECWs than others (e.g., see Tables 4 and 7); the CIs corresponding to bivariate *t*-distribution are generally wider than those corresponding to bivariate normal distribution; the ECWs decrease as the correlation coefficient *ρ* increases. Second, the RNCPs of all the considered CIs lie in the interval [0.4,0.6] (e.g., see Tables 5 and 8), which show that our derived CIs generally demonstrate symmetry. Third, when ${\sigma _{1}^{2}}={\sigma _{2}^{2}}$, the CIs based on statistics *T*_3_, *T*_4_ and *T*_5_ behave unsatisfactory (e.g., see Tables 9 and 10) because their corresponding ECPs are almost less than the pre-specified confidence level 95 %. Fourth, powers corresponding to *W*_*a*_ and *B*_1_ are larger than others (e.g., see Tables 12 and 13). From the above findings, we would recommend the usage of the Bootstrap-resampling-based CI (i.e., *B*_1_) because its coverage probability is generally close to the pre-chosen confidence level, it consistently yields the shortest interval width even when sample size is small, it usually guarantees its ratios of the MNCPs to the non-coverage probabilities lying in [0.4, 0.6], and its power is usually larger than others.

**Table 9:** ECPs of various confidence intervals with different *ρ* and *δ*, *μ*_1_, *μ*_2_, (*n*,*n*_1_,*n*_2_)=(5,5,2), when ${\sigma _{1}^{2}}={\sigma _{2}^{2}}=4$

Bivariate normal distribution												
*ρ*	*δ*	*μ*_1_	*μ*_2_	*T*_3_	*T*_4_	*T*_5_	*W*_*s*_	*W*_*a*_	*B*_1_	*B*_2_	*B*_3_	*B*_4_
-0.9	-0.25	0	0.25	0.935	0.960	0.906	0.920	0.880	0.952	0.954	0.947	0.954
	0	1	1	0.944	0.956	0.894	0.920	0.869	0.946	0.947	0.933	0.947
	0.5	2	1.5	0.944	0.967	0.902	0.931	0.883	0.951	0.953	0.942	0.951
-0.5	-0.25	0	0.25	0.941	0.961	0.903	0.910	0.861	0.942	0.943	0.939	0.943
	0	1	1	0.937	0.958	0.900	0.915	0.862	0.950	0.952	0.949	0.951
	0.5	2	1.5	0.941	0.962	0.898	0.925	0.882	0.952	0.957	0.952	0.957
-0.1	-0.25	0	0.25	0.933	0.958	0.900	0.903	0.838	0.944	0.945	0.945	0.946
	0	1	1	0.939	0.966	0.907	0.912	0.853	0.952	0.951	0.954	0.953
	0.5	2	1.5	0.943	0.975	0.924	0.943	0.892	0.961	0.959	0.960	0.959
0	-0.25	0	0.25	0.936	0.964	0.914	0.913	0.860	0.949	0.949	0.950	0.950
	0	1	1	0.925	0.959	0.906	0.908	0.861	0.941	0.941	0.940	0.940
	0.5	2	1.5	0.932	0.968	0.913	0.924	0.887	0.952	0.952	0.951	0.951
0.1	-0.25	0	0.25	0.922	0.960	0.918	0.911	0.858	0.948	0.948	0.948	0.947
	0	1	1	0.923	0.963	0.909	0.906	0.859	0.944	0.946	0.944	0.944
	0.5	2	1.5	0.928	0.969	0.913	0.935	0.889	0.946	0.947	0.947	0.946
0.5	-0.25	0	0.25	0.927	0.968	0.923	0.904	0.843	0.950	0.947	0.934	0.947
	0	1	1	0.928	0.964	0.923	0.913	0.857	0.942	0.944	0.935	0.947
	0.5	2	1.5	0.924	0.978	0.933	0.947	0.901	0.960	0.958	0.943	0.960
0.9	-0.25	0	0.25	0.913	0.947	0.974	0.929	0.880	0.951	0.951	0.777	0.951
	0	1	1	0.908	0.952	0.976	0.930	0.883	0.947	0.955	0.781	0.951
	0.5	2	1.5	0.913	0.942	0.974	0.974	0.944	0.946	0.953	0.778	0.954
Bivariate t-distribution												
-0.9	-0.25	0	0.25	0.922	0.972	0.908	0.929	0.870	0.952	0.953	0.946	0.956
	0	1	1	0.915	0.973	0.914	0.935	0.868	0.948	0.943	0.937	0.948
	0.5	2	1.5	0.930	0.978	0.914	0.937	0.873	0.948	0.950	0.941	0.951
-0.5	-0.25	0	0.25	0.929	0.976	0.921	0.939	0.869	0.942	0.941	0.940	0.945
	0	1	1	0.931	0.975	0.925	0.935	0.872	0.943	0.942	0.943	0.946
	0.5	2	1.5	0.922	0.971	0.910	0.924	0.868	0.953	0.951	0.950	0.955
-0.1	-0.25	0	0.25	0.932	0.973	0.922	0.925	0.856	0.951	0.951	0.955	0.954
	0	1	1	0.926	0.971	0.924	0.923	0.859	0.941	0.942	0.946	0.947
	0.5	2	1.5	0.924	0.972	0.918	0.921	0.859	0.950	0.948	0.954	0.955
0	-0.25	0	0.25	0.919	0.973	0.921	0.918	0.852	0.944	0.944	0.949	0.949
	0	1	1	0.925	0.972	0.923	0.925	0.864	0.940	0.940	0.947	0.947
	0.5	2	1.5	0.939	0.977	0.924	0.926	0.857	0.950	0.950	0.954	0.954
0.1	-0.25	0	0.25	0.930	0.971	0.929	0.928	0.857	0.954	0.954	0.956	0.956
	0	1	1	0.929	0.982	0.927	0.928	0.857	0.949	0.949	0.950	0.951
	0.5	2	1.5	0.934	0.979	0.924	0.930	0.859	0.952	0.953	0.957	0.957
0.5	-0.25	0	0.25	0.929	0.973	0.947	0.940	0.864	0.944	0.950	0.942	0.951
	0	1	1	0.920	0.976	0.937	0.928	0.861	0.943	0.944	0.936	0.946
	0.5	2	1.5	0.939	0.970	0.942	0.930	0.868	0.945	0.947	0.942	0.951
0.9	-0.25	0	0.25	0.923	0.969	0.978	0.943	0.880	0.939	0.938	0.797	0.939
	0	1	1	0.920	0.966	0.977	0.952	0.887	0.939	0.942	0.795	0.949
	0.5	2	1.5	0.931	0.965	0.979	0.944	0.878	0.953	0.944	0.804	0.947

**Table 10:** ECW of various confidence interals with different *ρ* and *δ*, *μ*_1_, *μ*_2_, (*n*,*n*_1_,*n*_2_)=(5,5,2), when ${\sigma _{1}^{2}}={\sigma _{2}^{2}}=4$

Bivariate normal distribution												
*ρ*	*δ*	*μ*_1_	*μ*_2_	*T*_3_	*T*_4_	*T*_5_	*W*_*s*_	*W*_*a*_	*B*_1_	*B*_2_	*B*_3_	*B*_4_
-0.9	-0.25	0	0.25	6.350	7.032	5.019	3.821	3.148	5.150	5.370	5.149	5.368
	0	1	1	6.389	7.038	5.047	3.833	3.162	5.151	5.370	5.151	5.370
	0.5	2	1.5	6.447	7.052	5.060	3.947	3.290	5.152	5.370	5.152	5.370
-0.5	-0.25	0	0.25	5.883	6.473	4.610	3.503	2.894	4.800	4.881	4.799	4.880
	0	1	1	5.885	6.436	4.606	3.510	2.903	4.800	4.881	4.799	4.879
	0.5	2	1.5	5.877	6.413	4.606	3.655	3.078	4.802	4.883	4.802	4.882
-0.1	-0.25	0	0.25	5.282	5.891	4.187	3.198	2.651	4.333	4.337	4.333	4.338
	0	1	1	5.318	5.898	4.186	3.213	2.670	4.335	4.340	4.334	4.338
	0.5	2	1.5	5.270	5.888	4.183	3.397	2.893	4.336	4.340	4.336	4.339
0	-0.25	0	0.25	5.114	5.733	4.046	3.096	2.571	4.190	4.190	4.189	4.189
	0	1	1	5.147	5.729	4.076	3.139	2.614	4.190	4.190	4.190	4.190
	0.5	2	1.5	5.123	5.763	4.069	3.337	2.849	4.191	4.191	4.189	4.189
0.1	-0.25	0	0.25	4.869	5.519	3.921	3.004	2.500	4.033	4.037	4.032	4.037
	0	1	1	4.870	5.550	3.899	3.004	2.504	4.032	4.037	4.033	4.038
	0.5	2	1.5	4.849	5.636	3.926	3.254	2.795	4.031	4.036	4.033	4.037
0.5	-0.25	0	0.25	3.805	5.050	3.412	2.608	2.188	3.202	3.360	3.202	3.360
	0	1	1	3.811	5.019	3.398	2.624	2.213	3.201	3.360	3.199	3.357
	0.5	2	1.5	3.857	5.211	3.401	2.955	2.583	3.200	3.359	3.200	3.360
0.9	-0.25	0	0.25	1.776	5.606	2.702	2.133	1.832	1.537	2.505	1.537	2.505
	0	1	1	1.766	5.561	2.676	2.147	1.853	1.539	2.503	1.538	2.503
	0.5	2	1.5	1.784	5.548	2.689	2.554	2.303	1.537	2.505	1.536	2.504
Bivariate t-distribution												
-0.9	-0.25	0	0.25	35.039	42.148	28.140	21.360	17.207	30.479	31.779	31.062	32.486
	0	1	1	35.226	42.660	28.523	21.569	17.374	30.470	31.763	31.048	32.470
	0.5	2	1.5	34.854	42.020	28.032	21.260	17.135	30.472	31.771	31.038	32.484
-0.5	-0.25	0	0.25	32.156	38.993	25.809	19.534	15.765	28.402	28.881	28.936	29.495
	0	1	1	33.177	39.103	26.338	19.953	16.106	28.417	28.901	28.961	29.518
	0.5	2	1.5	31.999	38.876	25.558	19.403	15.677	28.393	28.870	28.941	29.480
-0.1	-0.25	0	0.25	28.753	36.668	23.542	17.849	14.456	25.621	25.643	26.126	26.164
	0	1	1	28.672	36.649	23.652	17.809	14.435	25.637	25.661	26.146	26.184
	0.5	2	1.5	29.087	35.900	23.651	17.894	14.523	25.622	25.645	26.140	26.175
0	-0.25	0	0.25	27.123	35.382	22.633	17.113	13.892	24.786	24.786	25.284	25.284
	0	1	1	27.852	35.371	23.033	17.424	14.146	24.797	24.797	25.292	25.292
	0.5	2	1.5	27.607	34.434	22.581	17.116	13.919	24.786	24.786	25.288	25.288
0.1	-0.25	0	0.25	26.299	34.969	22.037	16.679	13.565	23.842	23.869	24.322	24.332
	0	1	1	26.797	35.384	22.411	16.960	13.787	23.854	23.882	24.349	24.365
	0.5	2	1.5	26.420	34.911	22.164	16.798	13.679	23.864	23.891	24.357	24.372
0.5	-0.25	0	0.25	20.192	32.428	19.137	14.443	11.860	18.938	19.877	19.369	20.262
	0	1	1	20.217	32.478	19.118	14.526	11.942	18.950	19.891	19.385	20.271
	0.5	2	1.5	20.314	30.975	18.783	14.325	11.783	18.928	19.869	19.361	20.257
0.9	-0.25	0	0.25	9.426	36.100	15.345	11.627	9.744	9.094	14.818	9.355	15.174
	0	1	1	9.491	34.843	15.055	11.622	9.750	9.090	14.804	9.352	15.167
	0.5	2	1.5	9.569	35.234	15.210	11.735	9.875	9.098	14.813	9.353	15.176

**Table 11:** RNCP of various confidence intervals with different *ρ* and *δ*, *μ*_1_, *μ*_2_, (*n*,*n*_1_,*n*_2_)=(5,5,2), when ${\sigma _{1}^{2}}={\sigma _{2}^{2}}=4$

Bivariate normal distribution												
*ρ*	*δ*	*μ*_1_	*μ*_2_	*T*_3_	*T*_4_	*T*_5_	*W*_*s*_	*W*_*a*_	*B*_1_	*B*_2_	*B*_3_	*B*_4_
-0.9	-0.25	0	0.25	0.4697	0.5652	0.4787	0.4000	0.5187	0.4583	0.5217	0.5185	0.5217
	0	1	1	0.4464	0.5968	0.4190	0.4304	0.4151	0.4815	0.4340	0.4478	0.4340
	0.5	2	1.5	0.4386	0.6170	0.4796	0.6324	0.4796	0.4898	0.4792	0.5000	0.4800
-0.5	-0.25	0	0.25	0.4915	0.5577	0.5258	0.4396	0.5258	0.5000	0.5088	0.5246	0.5088
	0	1	1	0.4444	0.5577	0.4800	0.4824	0.4800	0.4706	0.4286	0.4423	0.4490
	0.5	2	1.5	0.4915	0.5814	0.4950	0.6081	0.4902	0.4286	0.4545	0.4898	0.4773
-0.1	-0.25	0	0.25	0.4776	0.6042	0.4800	0.4330	0.4800	0.4912	0.4727	0.4630	0.4630
	0	1	1	0.4918	0.5714	0.4839	0.4773	0.4839	0.4583	0.4490	0.4783	0.4681
	0.5	2	1.5	0.5862	0.6563	0.5200	0.6724	0.5132	0.4750	0.4878	0.4878	0.4878
0	-0.25	0	0.25	0.5077	0.5641	0.5233	0.4598	0.5233	0.5385	0.5385	0.5600	0.5600
	0	1	1	0.5333	0.5769	0.5000	0.4891	0.5000	0.5085	0.5085	0.5000	0.5000
	0.5	2	1.5	0.5000	0.5957	0.5116	0.6053	0.5057	0.4167	0.4167	0.4286	0.4286
0.1	-0.25	0	0.25	0.5256	0.5652	0.5000	0.4205	0.5000	0.5000	0.5192	0.5000	0.5094
	0	1	1	0.4545	0.5625	0.4778	0.4681	0.4725	0.5179	0.5273	0.5179	0.5000
	0.5	2	1.5	0.5694	0.6486	0.5057	0.6212	0.5057	0.5741	0.5660	0.5556	0.5556
0.5	-0.25	0	0.25	0.5139	0.6604	0.4805	0.4167	0.4805	0.4510	0.4630	0.4615	0.4815
	0	1	1	0.4930	0.6667	0.5513	0.5057	0.5584	0.4746	0.5088	0.5077	0.5283
	0.5	2	1.5	0.5067	0.7027	0.5455	0.6604	0.5373	0.4878	0.5238	0.5439	0.5250
0.9	-0.25	0	0.25	0.5057	0.8286	0.5556	0.4028	0.5769	0.5000	0.4694	0.4798	0.4800
	0	1	1	0.4624	0.8333	0.5000	0.5000	0.5000	0.5185	0.4565	0.5227	0.5000
	0.5	2	1.5	0.4943	0.7733	0.4074	0.6538	0.4231	0.4630	0.5319	0.4775	0.5435
Bivariate t-distribution												
-0.9	-0.25	0	0.25	0.5195	0.6977	0.4891	0.5000	0.4930	0.4750	0.4375	0.4444	0.4318
	0	1	1	0.4706	0.6905	0.5349	0.5152	0.5231	0.4717	0.5690	0.5469	0.5769
	0.5	2	1.5	0.5362	0.7436	0.5000	0.5469	0.5556	0.5192	0.4800	0.5085	0.4898
-0.5	-0.25	0	0.25	0.5915	0.6818	0.4684	0.4426	0.4426	0.4915	0.5085	0.5000	0.5000
	0	1	1	0.4928	0.7143	0.4800	0.4531	0.4462	0.4912	0.4576	0.4737	0.4815
	0.5	2	1.5	0.5256	0.7021	0.5056	0.5526	0.5526	0.4167	0.3878	0.3529	0.3696
-0.1	-0.25	0	0.25	0.3971	0.5526	0.4937	0.4667	0.4667	0.5102	0.5000	0.5333	0.5217
	0	1	1	0.5270	0.7250	0.5395	0.5325	0.5325	0.4667	0.4655	0.4630	0.4717
	0.5	2	1.5	0.4605	0.5750	0.4444	0.4810	0.4810	0.5000	0.4717	0.5106	0.5000
0	-0.25	0	0.25	0.5309	0.6341	0.5000	0.4819	0.4878	0.5088	0.5088	0.4902	0.4902
	0	1	1	0.5067	0.6389	0.4805	0.4865	0.4800	0.5667	0.5667	0.5660	0.5660
	0.5	2	1.5	0.5574	0.7097	0.5132	0.5068	0.5000	0.5200	0.5200	0.5435	0.5435
0.1	-0.25	0	0.25	0.5714	0.5294	0.5556	0.5139	0.5139	0.5532	0.5652	0.5814	0.5814
	0	1	1	0.5211	0.7813	0.5833	0.5833	0.5833	0.5098	0.4902	0.5000	0.4898
	0.5	2	1.5	0.4925	0.6563	0.4800	0.5000	0.5000	0.5208	0.5106	0.5116	0.5116
0.5	-0.25	0	0.25	0.5493	0.6744	0.4717	0.4833	0.4833	0.4821	0.5000	0.5000	0.4800
	0	1	1	0.4625	0.7083	0.4444	0.4861	0.4861	0.4386	0.4912	0.4688	0.4630
	0.5	2	1.5	0.5161	0.6744	0.5172	0.5286	0.5286	0.5455	0.5283	0.5085	0.5306
0.9	-0.25	0	0.25	0.5455	0.8803	0.4348	0.5088	0.5088	0.4677	0.4677	0.4926	0.4754
	0	1	1	0.5570	0.8534	0.5652	0.6042	0.6042	0.5000	0.5000	0.5194	0.4706
	0.5	2	1.5	0.4348	0.8333	0.5714	0.4821	0.4821	0.5000	0.5357	0.4898	0.5370

**Table 12:** Power of various confidence intervals with different *ρ* and *δ*, *μ*_1_, $\mu _{2}, {\sigma _{1}^{2}}$ and (*n*,*n*_1_,*n*_2_)=(5,2,2) and ${\sigma _{2}^{2}}=4$

*ρ*	${\sigma _{1}^{2}}$	*δ*	*μ*_1_	*μ*_2_	*T*_1_	*T*_2_	*T*_*g*_	*W*_*s*_	*W*_*a*_	*B*_1_	*B*_2_	*B*_3_	*B*_4_
-0.9	1	-0.25	0	0.25	6.40	4.35	5.10	7.30	12.60	5.20	5.10	6.40	5.05
		0.5	2	1.5	7.25	5.20	5.50	9.10	13.70	6.50	6.35	7.80	6.35
	8	-0.25	0	0.25	5.80	3.30	5.25	7.60	13.30	5.50	5.10	6.10	4.95
		0.5	2	1.5	6.20	4.00	7.65	8.25	14.40	5.90	6.30	7.65	6.40
-0.5	1	-0.25	0	0.25	6.50	4.00	6.60	7.75	13.10	4.70	4.65	5.45	4.80
		0.5	2	1.5	7.75	4.90	7.60	10.15	15.85	6.10	5.95	6.40	5.70
	8	-0.25	0	0.25	6.60	4.55	7.00	9.55	15.30	5.80	5.40	6.20	5.70
		0.5	2	1.5	5.80	4.10	6.05	8.25	13.85	5.50	5.70	5.85	5.45
-0.1	1	-0.25	0	0.25	6.95	3.55	8.45	6.90	12.70	4.65	4.50	4.70	4.65
		0.5	2	1.5	7.70	4.85	7.55	9.90	15.75	6.80	6.80	6.85	6.65
	8	-0.25	0	0.25	7.25	3.95	7.90	9.75	15.60	6.25	6.15	6.25	6.20
		0.5	2	1.5	6.60	3.50	7.25	8.75	15.20	5.25	5.35	5.15	5.10
0	1	-0.25	0	0.25	8.10	4.60	7.10	8.20	13.40	5.45	5.45	5.45	5.45
		0.5	2	1.5	8.35	4.70	8.50	11.50	17.90	6.55	6.55	6.65	6.65
	8	-0.25	0	0.25	7.45	3.50	8.90	9.10	15.25	5.45	5.45	5.40	5.40
		0.5	2	1.5	7.30	3.65	7.45	10.55	16.80	6.10	6.10	6.10	6.10
0.1	1	-0.25	0	0.25	7.05	3.95	9.85	8.40	13.85	5.45	5.60	5.60	5.70
		0.5	2	1.5	7.55	4.45	8.45	11.55	16.90	5.85	6.15	5.90	5.95
	8	-0.25	0	0.25	6.30	3.85	8.70	8.05	14.20	4.75	4.85	5.00	5.05
		0.5	2	1.5	7.65	4.05	9.60	9.70	16.40	5.85	6.00	6.25	6.30
0.5	1	-0.25	0	0.25	7.30	4.15	9.35	6.95	12.90	5.10	4.85	6.15	4.90
		0.5	2	1.5	8.40	4.75	8.15	12.70	19.35	6.00	5.95	7.10	6.15
	8	-0.25	0	0.25	8.80	4.20	7.80	9.80	15.40	5.30	5.15	6.80	5.30
		0.5	2	1.5	9.10	4.05	8.40	11.55	16.45	6.65	6.95	8.50	7.15
0.9	1	-0.25	0	0.25	7.30	5.25	8.10	7.50	13.60	5.10	5.35	7.20	5.40
		0.5	2	1.5	8.45	5.35	8.55	18.00	26.95	7.55	7.70	8.25	7.75
	8	-0.25	0	0.25	8.95	5.40	5.35	7.25	13.45	5.80	5.90	7.10	6.10
		0.5	2	1.5	11.45	5.30	6.25	12.30	18.20	10.05	8.00	9.60	7.95

**Table 13:** Power of various confidence intervals with different *ρ* and *δ*, *μ*_1_, *μ*_2_, (*n*,*n*_1_,*n*_2_)=(5,5,2), when ${\sigma _{1}^{2}}={\sigma _{2}^{2}}=4$

Bivariate normal distribution												
*ρ*	*δ*	*μ*_1_	*μ*_2_	*T*_3_	*T*_4_	*T*_5_	*W*_*s*_	*W*_*a*_	*B*_1_	*B*_2_	*B*_3_	*B*_4_
-0.9	-0.25	0	0.25	1.5	2.5	4.3	7.5	12.4	5.2	4.8	6.6	5.0
	0.5	2	1.5	3.2	4.1	6.4	10.8	15.6	7.5	7.4	9.4	7.4
-0.5	-0.25	0	0.25	3.9	3.0	5.7	8.5	12.9	5.6	5.1	5.6	5.2
	0.5	2	1.5	4.0	3.0	6.5	9.9	14.4	6.8	6.9	7.3	6.8
-0.1	-0.25	0	0.25	3.6	2.9	6.2	9.5	14.8	5.8	5.8	5.9	6.0
	0.5	2	1.5	5.5	4.9	8.7	11.3	16.4	8.3	8.2	7.9	7.9
0	-0.25	0	0.25	4.4	3.3	6.9	9.8	14.7	5.7	5.7	5.9	5.9
	0.5	2	1.5	4.7	4.0	7.6	10.8	16.7	7.9	7.9	7.6	7.6
0.1	-0.25	0	0.25	3.5	2.9	5.5	8.2	13.3	5.8	5.7	5.7	5.7
	0.5	2	1.5	5.1	4.3	8.1	11.6	16.2	7.6	7.3	7.5	7.4
0.5	-0.25	0	0.25	4.7	3.3	5.9	9.6	14.7	6.7	6.5	8.5	6.3
	0.5	2	1.5	5.3	5.1	8.4	13.1	17.9	11.1	10.8	13.2	10.6
0.9	-0.25	0	0.25	3.9	3.5	4.7	9.7	15.4	10.7	6.5	27.5	6.4
	0.5	2	1.5	9.1	6.0	8.2	13.7	18.0	27.9	11.4	27.3	11.2
Bivariate t-distribution												
-0.9	-0.25	0	0.25	1.2	2.1	4.0	6.7	11.6	4.9	5.1	5.9	4.7
	0.5	2	1.5	1.5	2.0	4.0	6.1	11.4	4.9	5.0	6.1	4.3
-0.5	-0.25	0	0.25	2.0	1.5	4.2	6.2	12.2	4.8	5.1	5.1	4.9
	0.5	2	1.5	2.0	1.8	5.0	6.8	12.7	6.3	6.3	6.4	5.9
-0.1	-0.25	0	0.25	2.9	2.8	6.0	8.3	15.2	7.1	7.0	6.7	6.4
	0.5	2	1.5	2.0	1.9	5.0	7.0	12.7	4.4	4.4	4.1	4.0
0	-0.25	0	0.25	2.5	2.0	4.1	6.7	12.4	5.0	5.0	4.5	4.5
	0.5	2	1.5	2.2	1.9	4.6	6.5	12.8	6.1	6.1	5.9	5.9
0.1	-0.25	0	0.25	2.4	2.1	4.4	7.0	12.0	5.2	5.1	5.0	5.0
	0.5	2	1.5	2.9	2.7	5.6	7.4	13.2	5.3	5.1	4.9	5.0
0.5	-0.25	0	0.25	1.3	2.0	4.4	6.1	11.4	5.0	5.2	6.4	5.2
	0.5	2	1.5	1.7	2.0	4.7	6.1	11.4	4.9	5.1	5.9	4.7
0.9	-0.25	0	0.25	1.3	2.8	3.4	5.0	10.4	5.0	4.8	5.4	4.4
	0.5	2	1.5	2.1	2.2	2.7	5.1	11.7	5.7	5.8	5.8	5.2

### An worked example

In this subsection, the data introduced in Section for the action of two doses of formoterol solution aerosol are used to illustrate the proposed methodologies. In this example, we are interested in CI construction of the difference of two FEV_1_ values for two doses of formoterol solution aerosol. Under the previously given notation, we have *n*=7, *n*_1_=9, *n*_2_=8, $\hat \delta =a\overline {x}_{1}^{(n)}+\left (1-a\right)\overline {x}_{1}^{(n_{1})}-b\overline {x}_{2}^{(n)}-(1-b)\overline {x}_{2}^{(n_{2})}=-0.0840$ (or $\hat {\delta }=\sum _{j=1}^{n+n_{1}}x_{1j}/(n+n_{1})-\sum _{j=1}^{n+n_{2}}x_{2j}/(n+n_{2})=0.0228$). Various 95 % CIs for *δ* under *Σ* unknown assumption are presented in Table 14. Examination of Table 14 shows that the actions of two doses of formaterol solutions aerosol are the same because all the derived CIs include zero.

**Table 14:** Various 95 % confidence intervals for *δ*=*μ*_1_−*μ*_2_ based on formoterol solution aerosol

	*T*_1_	*T*_2_	*T*_3_	*T*_4_	*T*_5_	*T*_*g*_
Lower	-0.2751	-0.4764	-0.472	-0.5542	-0.4431	-0.4883
Upper	0.1071	0.5220	0.3741	0.5999	0.4888	0.5039
Width	0.3822	0.9984	0.8461	1.1541	0.9319	0.9922
	*W*_*s*_	*W*_*a*_	*B*_1_	*B*_2_	*B*_3_	*B*_4_
Lower	-0.5940	-0.5787	-0.5408	-0.5938	-0.5259	-0.5681
Upper	0.6495	0.6334	0.3995	0.4394	0.4309	0.4058
Width	1.2435	1.2121	0.9403	1.0332	0.9568	0.9739

## Discussion

Although testing equivalence of two correlated means with incomplete data has been studied, there is little work done on their interval estimators. To address the issue, this paper proposes various interval estimators of the difference of two correlated means for *Σ* known and unknown cases based on the large sample method, hybrid method and Bootstrap-resampling method. Extensive simulation studies are conducted to evaluate the finite performance of the proposed CIs in terms of the empirical coverage probability, empirical interval width and ratio of the mesial non-coverage probability to the non-coverage probability (RNCP). Empirical results evidence that the Bootstrap-resampling-based CIs *B*_1_, *B*_2_, *B*_4_ behave satisfactorily for small to moderate sample sizes in the sense that their coverage probabilities could be well controlled around the pre-specified nominal confidence level and their RNCPs almost lie in the interval [0.4, 0.6]. However, confidence intervals based on the large sample method and hybrid method behave unsatisfactory for small sample sizes because the distributions of statistics *T*_1_,⋯,*T*_5_ are asymptotical, and these asymptotical distributions are proper only when *N*_*i*_→*∞*. When *Σ* is unknown, using GEE method to estimate variance is less efficient.

It is interesting to investigate confidence interval construction of the difference of two means with incomplete correlated data under missing at random and non-ignorable missing data mechanism assumptions of bivariate variables. We are working on the topics.

## Conclusion

According to the aforementioned findings, we can draw the following conclusions. The Bootstrap-resampling-based CI *B*_1_ is a desirable interval estimator for the difference of two means with incomplete correlated data.
